# Comparative chloroplast genomics: analyses including new sequences from the angiosperms *Nuphar advena *and *Ranunculus macranthus*

**DOI:** 10.1186/1471-2164-8-174

**Published:** 2007-06-15

**Authors:** Linda A Raubeson, Rhiannon Peery, Timothy W Chumley, Chris Dziubek, H Matthew Fourcade, Jeffrey L Boore, Robert K Jansen

**Affiliations:** 1 Biological Sciences, Central Washington University, Ellensburg, WA 98926-7537, USA; 2 Section of Integrative Biology and Institute of Cellular and Molecular Biology, The University of Texas at Austin, Austin, TX 78712, USA; 3 DOE Joint Genome Institute and Lawrence Berkeley National Laboratory, Program in Evolutionary Genomics, Walnut Creek, CA 94547, USA

## Abstract

**Background:**

The number of completely sequenced plastid genomes available is growing rapidly. This array of sequences presents new opportunities to perform comparative analyses. In comparative studies, it is often useful to compare across wide phylogenetic spans and, within angiosperms, to include representatives from basally diverging lineages such as the genomes reported here: *Nuphar advena *(from a basal-most lineage) and *Ranunculus macranthus *(a basal eudicot). We report these two new plastid genome sequences and make comparisons (within angiosperms, seed plants, or all photosynthetic lineages) to evaluate features such as the status of *ycf15 *and *ycf68 *as protein coding genes, the distribution of simple sequence repeats (SSRs) and longer dispersed repeats (SDR), and patterns of nucleotide composition.

**Results:**

The *Nuphar *[GenBank:NC_008788] and *Ranunculus *[GenBank:NC_008796] plastid genomes share characteristics of gene content and organization with many other chloroplast genomes. Like other plastid genomes, these genomes are A+T-rich, except for rRNA and tRNA genes. Detailed comparisons of *Nuphar *with *Nymphaea*, another Nymphaeaceae, show that more than two-thirds of these genomes exhibit at least 95% sequence identity and that most SSRs are shared. In broader comparisons, SSRs vary among genomes in terms of abundance and length and most contain repeat motifs based on A and T nucleotides.

**Conclusion:**

SSR and SDR abundance varies by genome and, for SSRs, is proportional to genome size. Long SDRs are rare in the genomes assessed. SSRs occur less frequently than predicted and, although the majority of the repeat motifs do include A and T nucleotides, the A+T bias in SSRs is less than that predicted from the underlying genomic nucleotide composition. In codon usage third positions show an A+T bias, however variation in codon usage does not correlate with differences in A+T-richness. Thus, although plastome nucleotide composition shows "A+T richness", an A+T bias is not apparent upon more in-depth analysis, at least in these aspects. The pattern of evolution in the sequences identified as *ycf15 *and *ycf68 *is not consistent with them being protein-coding genes. In fact, these regions show no evidence of sequence conservation beyond what is normal for non-coding regions of the IR.

## Background

In this paper we report the complete chloroplast genome sequences of the angiosperms *Nuphar advena *(Nymphaeaceae) and *Ranunculus macranthus *(Ranunculaceae). The Nymphaeaceae is placed very near or even at the base of extant angiosperms [1-6], whereas the *Ranunculus *chloroplast genome represents the basal-most eudicot characterized to date [4,5]. Thus we add to the small number of genomes not representing monocots or crown eudicots, increasing our ability to compare genomes across all angiosperms and determine general characteristics. Most chloroplast genomes in angiosperms (reviewed in: [7-9]) range from 135 to 160 kb and exist, at least in part [10] as single genome circles. In the majority of angiosperm chloroplast genomes two copies of a large inverted repeat (IR) of about 25 kb separate the remainder of the genome into two regions of unique DNA, the large (about 90 kb) and small (about 20 kb) single copy regions (LSC and SSC, respectively). Tobacco (*Nicotiana tabacum*), the first completely sequenced chloroplast genome [11], is most frequently used to contrast features of newly studied angiosperm cpDNAs and is parsimoniously inferred to represent the ancestral (or at least consensus) angiosperm chloroplast genome in terms of gene content and organization [7,8]. This is reinforced by the similarity of the tobacco cpDNA to the basal angiosperms, *Amborella *[12] and *Nymphaea *[13], and the magnolids, *Calycanthus *[14]*Drimys, Liriodendron*, and *Piper *[6]. In some derived angiosperm lineages, this ancestral condition has been somewhat or highly modified via inversions, gene losses, presence or absence of ORFs and minor ycfs, and changes in IR extent [8,9,15,16]. However, the tobacco-like pattern is widely distributed in crown eudicots, e.g., *Panax *[17], *Eucalyptus *[18], and *Gossypium *[19].

Comparative chloroplast genomics as well as detailed characterizations of individual chloroplast genomes serve as the basis for functional genomic studies [20] and can direct attempts at chloroplast transformation for genetic engineering [21]. In addition the chloroplast genome is an important source of genetic markers for phylogenetic analysis, population-level studies, genotyping and mapping that can be further exploited with additional genomic characterization and comparison. Detailed comparisons of genomic sequence have the potential, for example, to identify functional sequence outside of coding regions (promoters, terminators, replication origins, etc.), test the reality of hypothetical protein coding regions, make inferences about mutational rates and mechanisms, and detect selective signatures in gene sequences. Many fundamental aspects of the chloroplast genome are poorly understood and incompletely described. Here we use genomic comparisons to investigate the likelihood that *ycf15 *and *ycf68 *are not protein-coding genes, the occurrence of microsatellites or simple sequence repeats (SSRs), the presence of somewhat larger more complex repeats or small disperse repeats (SDR) and how nucleotide composition contributes to patterns of genome organization such as codon usage and repeat structure.

## Results and Discussion

### Genomic characteristics, including IR extent

**Figure 1:**
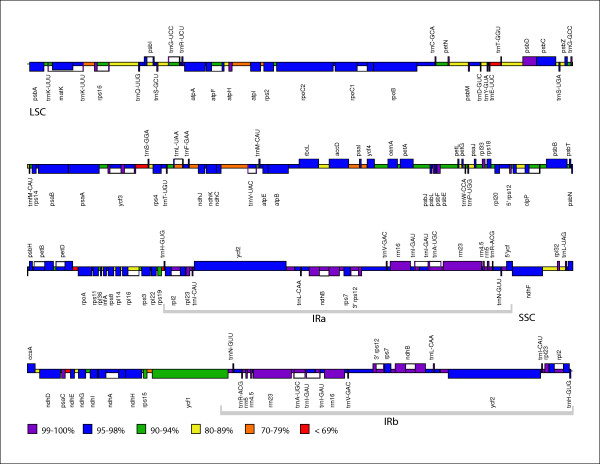
Linearized *Nuphar advena *plastome map. Genes are represented by boxes extending above or below the base line depending on the direction of transcription. The color of the gene boxes and the intergenic regions indicates the level of similarity of the region between the *Nuphar *and *Nymphaea *plastomes.

**Figure 2:**
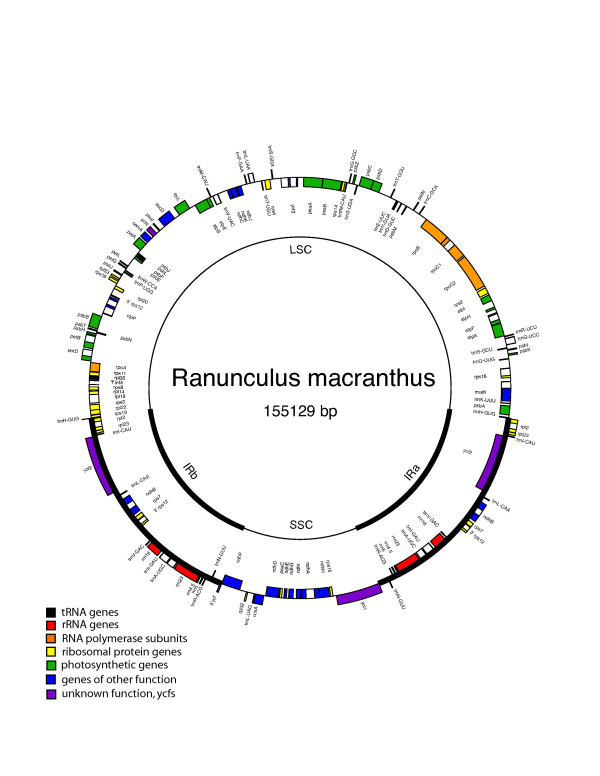
Circular *Ranunculus macranthus *plastome map. Genes are represented by boxes inside or outside the circle to indicate the direction of transcription, clockwise or counterclockwise, respectively. The color of the gene boxes indicates the functional group to which the gene belongs.

Both the *Nuphar *[GenBank:NC_008788] and *Ranunculus *[GenBank:NC_008796] genomes exhibit the quadripartite structure common to most land plant genomes with large and small single copy regions (LSC and SSC, respectively) separated by two copies of the IR. The *Nuphar *chloroplast genome (Figure 1) is 160,866 bp in total length; the LSC is 90,379 bp, the SSC 18,817 bp and the two IR copies each 25,835 bp in length. In *Ranunculus *(Figure 2), the overall length is 155,129 bp with a LSC of 84,638 bp, a SSC of 18,909 bp, and two IR regions each of 25,791 bp. As is common to chloroplast genomes in general [5,6,9], the nucleotide composition of both of these genomes are biased towards A and T nucleotides, i.e., they are "A+T-rich". Overall the *Nuphar *genome is 60.9% A+T and *Ranunculus *62.1% A+T. Different regions of the genome vary in A+T content, but all partitions are A+T rich with the exception of the two classes of RNA genes (Table 1).

**Table 1:** AT richness (%A+T) of the *Nuphar *and *Ranunculus *plastomes, overall and in various partitions.

	Genome	LSC	SSC	IR	Non-coding (IGS)	Protein- coding genes (CDS)
*Nuphar*	60.9	62.9	65.3	56.7	65.5	60.6
*Ranunculus*	62.1	65.5	69.0	56.5	68.3	62.0

	introns	1st position	2nd position	3rd position	rRNA genes	tRNA genes

*Nuphar*	63.0	53.1	60.8	68.0	44.4	46.5
*Ranunculus*	63.5	54.2	61.7	70.1	44.5	46.8

Although the two copies of the IR were not sequenced independently, the identity of the two copies could still be assessed. All random reads generated from the two IR regions falsely assemble in one location, but presumably about half of the reads are sequenced from one copy of the repeat and half from the other. If the two copies varied in sequence, one would expect, on average, half the reads to reflect one variant and half the other, but all reads that assembled into this region for both *Nuphar *and *Ranunculus *were identical in sequence. In fact, throughout the entire cpDNA of *Nuphar*, only one case of potential heteroplasmy was observed, where four reads indicated a run of 10 As and four others indicated 11. In *Ranunculus *no cases of high quality mismatch involving more than a single read were detected, even though multiple individuals contributed to the sequencing template. Nucleotide polymorphisms in chloroplast DNA sequences have been detected in several other groups. In the completely sequenced *Pelargonium *genome [16], 11 polymorphisms were detected and nine of these were located in the LSC. Unfortunately, it was not possible to determine if the differences in *Nuphar *or *Pelargonium *represent heteroplasmy because multiple individuals were used in both studies. Heteroplasmy in the chloroplast genome has been detected in several other groups, including rice [22], *Medicago *[23], and *Senecio *[24].

**Figure 3:**
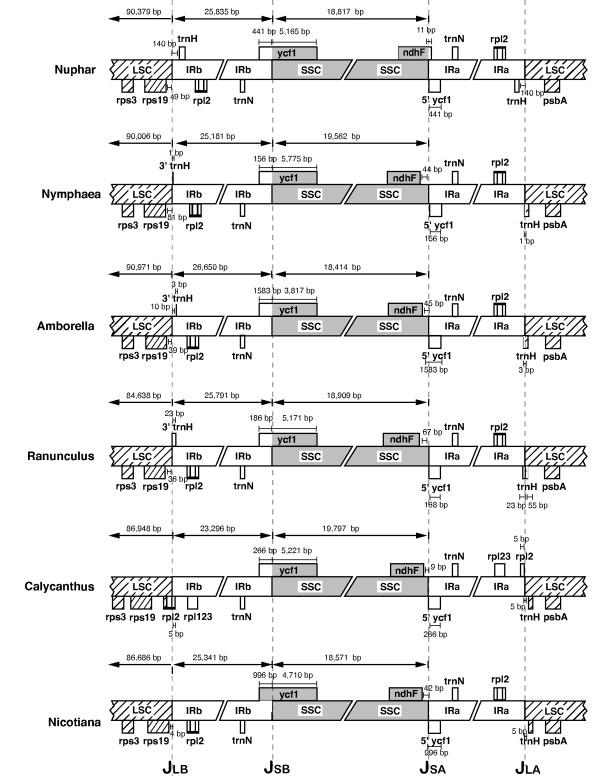
Comparison of inverted repeat-single copy boundaries in six representative angiosperms. Variation occurs at each of the four junctions. In *Calycanthus rpl2 *is not in the IR. J_SB _occurs within *ycf1 *in all of the genomes but the amount of the 5' end of *ycf1 *that is duplicated ranges from 156 bp in *Nymphaea *to 1583 bp in *Amborella*. Eleven bp of *ndhF *is duplicated in *Nuphar *but none of the other genomes shown have any duplication of the gene. J_LA _varies from including 5 bp of spacer downstream of *trnH *in *Nicotiana *to the inclusion of *trnH *and an additional 140 bp upstream sequence in the IR in *Nuphar*.

The IR extent in *Nuphar *and *Ranunculus*, as well as those of the basal angiosperms sequenced by Goremykin et al [12-14], were confirmed independently of the primary sequencing effort by PCR amplification and sequencing of the boundary regions. Although all these genomes contain IRs similar in extent to those of *Nicotiana *and many other angiosperms, some minor modifications were detected (Figure 3). As is common among angiosperms, a complete copy of *ycf1 *spans the SSC/IR_A _junction and the 5'end of the gene is duplicated at the SSC end of IRb. Among the comparisons shown in Figure 3, the amount of *ycf1 *that is duplicated ranges from 156 bp in *Nymphaea *to 1,583 bp in *Amborella*. At the LSC end of the IR, there is variation in these taxa over whether and how much of the gene *trnH *is duplicated. No part of *trnH *is duplicated in *Calycanthus *or *Nicotiana *but amounts ranging from one bp in *Nymphaea *to the entire gene (plus 140 bp of IGS) in *Nuphar *have been incorporated into the IR. The *trnH *gene has also been incorporated in the IR of *Drimys *but not in the other two recently sequenced magnoliid genomes, *Liriodendron*, and *Piper *[6]. Large or complex changes in the extent of the IR should make distinctive markers of evolutionary lineages [25,26]. However, given the small size of the changes discussed here and the relative ease of small (~100 bp) movements of the IR boundaries [27], the amount of *ycf1 *duplicated and the migration of *trnH *relative to the IR would not make very reliable phylogenetic markers.

### Gene content, including ycf15 and ycf68

The gene content and arrangement of *Nuphar *and *Ranunculus *plastid genomes (Figs. 1, 2) are identical with each other and with *Nymphaea*, *Calycanthus*, *Amborella *and *Nicotiana *(among others) except for details of the IR extent (described above), whether or not *infA *occurs as a pseudogene (in *Ranunculus *and *Nicotiana*) or a functional copy (the others listed), and the nature of *ycf15 *and *ycf68 *(see below). Seventy-nine different protein-coding genes (including, in this count, four hypothetical genes, *ycf1*, *ycf2*, *ycf3*, and *ycf4*), four rRNA genes and 30 tRNA genes occur in these genomes. Eighteen of these genes contain introns including two genes, *clpP *and *ycf3*, each with two introns, and one gene, *rps12*, also composed of three exons, but with the 5' exon separated from the two 3' exons. These features are common characteristics of land plant chloroplast genomes [9,28].

**Figure 4:**
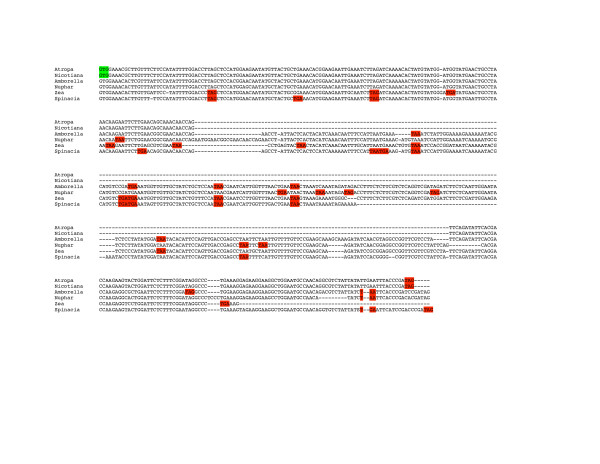
Alignment of the *ycf15 *region in six representative angiosperms. *Atropa *and *Nicotiana *represent the uninterrupted form. Codons highlighted in green represent start codons as annotated in the published genomes *Atropa *and *Nicotiana*. Codons highlighted in red represent stop codons in frame with those start codons. Although the sequence is highly conserved, it is not an open reading frame in most taxa.

The hypothetical gene *ycf15 *was first identified as ORF87 in *Nicotiana *[11] and has been included in the annotation of a subset of the completed land plant genomes. However, the validity of *ycf15 *as a protein-coding gene has been questioned [14,18,29]. Schmitz-Linneweber et al [29] found that the plastomes of *Nicotiana*, *Epifagus *and *Cuscuta *contain intact copies of *ycf15*, whereas those of *Spinacia *and *Arabidopsis *contain *ycf15 *as two pieces, with the 5' and 3' sections separated by 250–300 bp of 'intervening sequence'. They reasoned that if *ycf15 *is a functional protein in spinach, then the intervening sequence would need to be removed and the 5' and 3' sections spliced in order to make a functional *ycf15 *mature transcript. If the intervening sequence were not removed, numerous in-frame stop codons would lead to a truncated protein (Figure 4). Reverse transcription experiments in spinach determined that *ycf15 *was not spliced (although it was transcribed) and so presumably was not translated in spinach [29]. They concluded that the *ycf15 *sequence, since it is highly conserved, probably has functional significance but probably does not code for a protein. Using an alternative approach, Goremykin et al [14] compared nucleotide substitution rates (dN/dS) in *ycf15 *and found a ratio that suggests *ycf15 *is not a protein evolving under evolutionary constraint.

We examined the *ycf2:trnL *spacer region, where *ycf15 *is located, in several chloroplast genomes, including *Nuphar *and *Ranunculus*, to determine the distribution and nature of *ycf15 *(Table 2). Schmitz-Linneweber *et al*. [29], based on their small sample of taxa, suggested that the distribution of the interrupted versus uninterrupted *ycf15 *sequence, whether or not the sequence codes for a protein, might have phylogenetic significance. That indeed appears to be the case; all taxa, among those we examined, that contain an uninterrupted *ycf15 *motif are asterids and no asterid has the interrupted form (although some lack the 5' portion of the motif). We assume, based on the sequence similarity of the intervening sequence, that the Schmitz-Linneweber *et al*. [29] finding of lack of splicing in *Spinacia *is likely to hold for other interrupted forms of the motif. The existence of the widely distributed interrupted form suggests that this is not a protein-coding gene in any of these genomes. If this truly is a protein-coding gene in the asterids, we would have to imagine that pseudogenes (as the interrupted forms would be, see Figure 4) are being retained, conserved over broad evolutionary distances, and either that the active form was inactivated multiple times by the insertion of the same intervening sequence at the same location or that an inactivated gene was re-activated in the asterids through the excision of the intervening sequence. Thus, we concur that *ycf15 *is unlikely to represent a protein-coding gene and so we did not annotate the sequence in either genome.

**Table 2:** Extent of *ycf15 *motif (as defined by the tobacco sequence) in published angiosperm chloroplast genomes.

	Species^a^	Accession Number	5' portion	3' portion	Length of intervening sequence^b ^(bp)
"basal" angiosperms	*Amborella trichopoda*	NC_005086	154	92	295
	*Nuphar advena*	NC_008788	154	70	299
	*Nymphaea alba*	NC_006050	154	74	299
	*Calycanthus floridus*	NC_004993	152	92	291
Monocots	*Acorus calamus*	NC_007407	154	0	N/A
	*Phalaenopsis aphrodite*	NC_007499	136	45	295
	*Saccharum officinarum*	NC_006084	139	79	305
	*Zea mays*	NC_001666	139	79	305
Eudicots Non-asterids	*Arabidopsis thaliana*	NC_000932	120	105	285
	*Eucalyptus globulus*	NC_008115	154	106	296
	*Gossypium hirsutum*	NC_007944	154	107	307
	*Lotus japonicus*	NC_002694	0	98	N/A
	*Oenothera elata*	NC_002693	154	110	284
	*Ranunculus macranthus*	NC_008796	0	78	N/A
	*Spinacia oleracea*	NC_002202	153	92	254
	*Vitis vinifera*	NC_007957	154	107	292
Eudicots Asterids	*Atropa belladonna*	NC_004561	154	110	0
	*Epifagus virginiana*	NC_001568	150	107	0
	*Helianthus annuus*	NC_007977	0	53	N/A
	*Lactuca sativa*	DQ383816	0	53	N/A
	*Lycopersicon esculentum*	DQ347959	154	110	0
	*Nicotiana sylvestris*	NC_007500	154	110	0
	*Nicotiana tobaccum*	NC_001879	154	110	0
	*Nicotiana tomentosiformis*	NC_007602	154	110	0
	*Panax ginseng*	NC_006290	154	106	0
	*Solanum bulbocastanum*	NC_007943	154	110	0
	*Solanum tuberosum*	NC_008096	154	110	0

^a ^*ycf15 *is 264 bp in the original annotation of *Nicotiana*, 147 bp in *Amborella*, 243 bp in *Nymphaea *and *Calycanthus*, 300 bp in 234 bp in *Arabidopsis*, 138 bp in *Gossypium*, 234 bp in *Vitus*, 213 bp in *Atropa*, 264 bp in *Nicotiana sp*. and *Solanum sp*., 264 bp in *Lycopersicon*, 162 bp in *Helianthus*, and 303 bp in *Panax*.

^b ^N/A = not applicable (cases where either 5' or 3' portion is missing)

**Figure 5:**
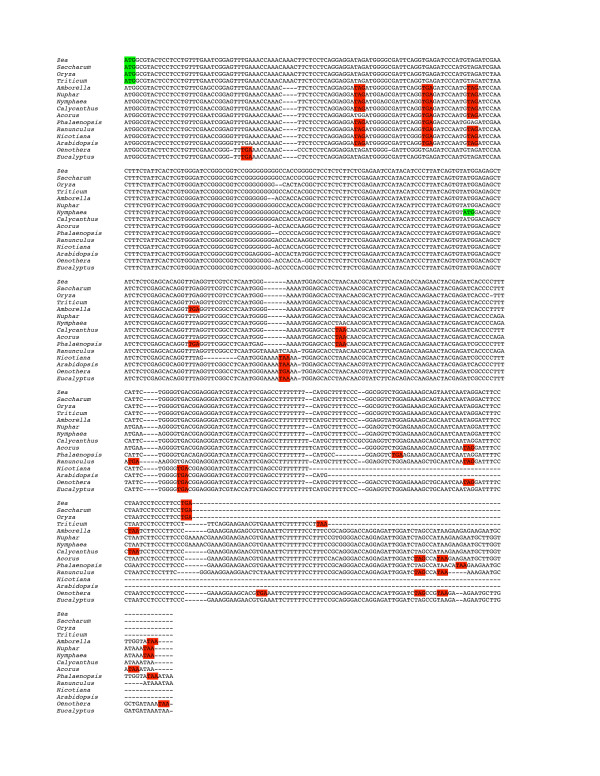
Alignment of the *ycf68 *region in 14 representative angiosperms. *Amborella, Nuphar, Zea*, and *Spinacia *represent the form that includes intervening sequence. Codons highlighted in green represent start codons as annotated in the published grass genomes (*Zea*, *Saccharum*, *Oryza *and *Triticum*) and *Nymphaea*. Gorymekin et al identified a later start codon in their annotation of the Nymphaea *ycf68 *in order to maintain an open reading frame. Codons highlighted in red represent in frame stop codons (in frame with the grass start codon in the initial part of the alignment and in frame with the *Nymphaea *start codon once that point is reached). In either frame, these sequences, although largely conserved at the nucleotide level, are not open in most taxa.

Similarly a second hypothetical protein-coding gene, *ycf68*, also may not code for a protein. This conserved motif has been reported in the *trnI*-GAU intron of rice (ORF133), corn (ORF133), *Pinus *(ORF75a), *Eucalyptus *(ORF113) and *Nymphaea*. Wheat and sugarcane also contain an ORF in this region apparently homologous to that of rice and corn [30]. We did not find an equivalent sequence in the chloroplast genome of any alga, which all lack an intron in the *trnI*-GAU gene, or in *Selaginella*, which lacks the *trnI*-GAU gene (Table 3). In the plastid genomes of all other vascular plant taxa examined, a similar sequence occurs in the *trnI *intron but in the majority of cases it contains numerous frameshifts and stop codons (Figure 5). Based only on its length and lack of internal stop codons, the *ycf68 *sequence could represent a functional protein-coding gene in the grasses, Nymphaeales, and in the gymnosperms *Pinus thunbergii *and *P. koraiensis*. However, in the others it can only be, at most, a pseudogene. Again, it seems unlikely that a non-functional gene could remain as conserved as the motif seen here over such vast evolutionary distances. If the sequence has any functional significance it must be other than coding for a protein, for example, in intron excision or in gene regulation. We did attempt to detect relationships between the conserved *ycf68 *motif and folding of the intron without success (see methods). We did not include *ycf68 *in the annotation of either *Nuphar *or *Ranunculus*.

**Table 3:** Distribution of the *ycf68 *motif in completely sequenced chloroplast genomes.

Species	Accession number	Absent^a^	Present but cannot be functional^b^	Present without internal stops^c^
*Nuphar advena*	NC_008788			102 aa
*Nymphaea alba*	NC_006050			102 aa
*Oryza sativa*	NC_001320			134 aa
*Pinus thunbergii*	NC_001631			75 aa
*Saccharum officinarum*	NC_006084			134 aa
*Triticum aestivum*	NC_002762			144 aa
*Zea mays*	NC_001666			134 aa
*Acorus calamus*	NC_007407		X	
*Adiantum capillus-veneris*	NC_004766		X	
*Amborella trichopoda*	NC_005086		X	
*Anthocerus formosae*	NC_004543		X	
*Arabidopsis thaliana*	NC_000932		X	
*Atropa belladonna*	NC_004561		X	
*Calycanthus floridus*	NC_004993		X	
*Cucumis sativus*	NC_007144		X	
*Epifagus virginiana*	NC_001568		X	
*Eucalyptus globulus*^d^	NC_008115		X	
*Huperzia lucidula*	NC_006861		X	
*Lactuca sativa*	NC_007578		X	
*Lotus japonicus*	NC_002694		X	
*Marchantia polymorpha*	NC_001319		X	
*Medicago polymorpha*	NC_003119		X	
*Nicotiana tabacum*	NC_001879		X	
*Oenothera elata*	NC_002693		X	
*Panax ginseng*	NC_006290		X	
*Phalaenopsis aphrodite*	NC_007499		X	
*Physcomitrella patens*	NC_005087		X	
*Psilotum nudum*	NC_003386		X	
*Ranunculus macranthus*	NC_008796		X	
*Spinacia oleracea*	NC_002202		X	
*Chaetosphaeridium globosum*	NC_004115	X^e^		
*Chlamydomonas reinhardtii*	NC_005353	X^e^		
*Chlorella vulgaris*	NC_001865	X^e^		
*Cyanidioschyzon merolae*	NC_004799	X^e^		
*Cyanidium caldarium*	NC_001840	X^e^		
*Cyanophora paradoxa*	NC_001675	X^e^		
*Eimeria tenella*	NC_004823	X^e^		
*Emiliania huxleyi*	NC_007288	X^e^		
*Euglena gracilis*	NC_001603	X^e^		
*Gracilaria tenuistipitata*	NC_006137	X^e^		
*Guilardia theta*	NC_000926	X^e^		
*Mesostigma viride*	NC_002186	X^e^		
*Nephroselmis olivacea*	NC_000927	X^e^		
*Odontella sinensis*	NC_001713	X^e^		
*Porphyra purpurea*	NC_000925	X^e^		
*Selaginella uncinata*	NC_007625	X^f^		
*Toxoplasma gondii*	NC_001799	X^e^		

^a ^No significant similarity in blast search to published ycf68 regions anywhere in genome

^b ^A complete copy of the motif is present but there are internal stops making it "non-functional"

^c ^*ycf68 *motif occurs as ORF; Recognized as CDS in the genome annotation

^d ^Published *Eucalyptus *genome map [18] includes *ycf68*, but *ycf68 *region includes internal stops

^e^no intron in *tRNA*-Ile

^f^no *tRNA*-Ile

**Figure 6:**
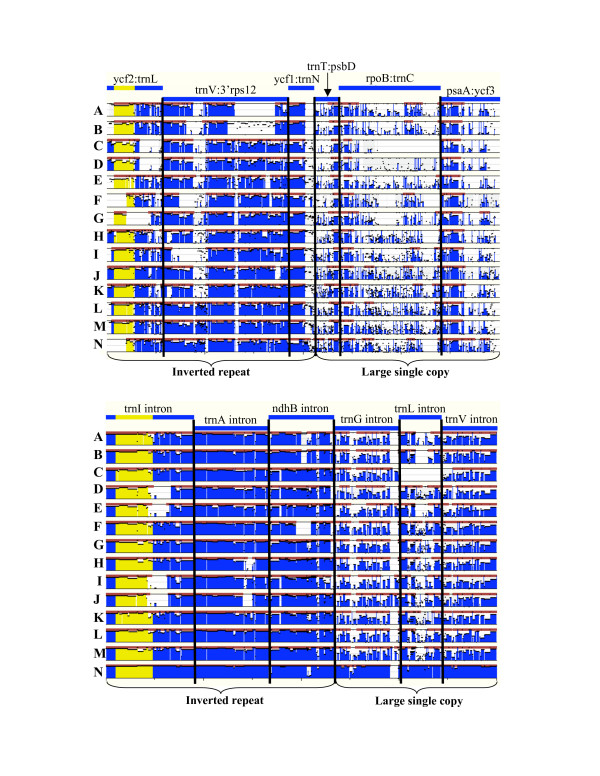
Sequence similarity comparisons of IGS and introns within the IR and the LSC. In both the top and bottom section, each 14 pairwise Mulan alignments is displayed as a histogram showing the similiarity (ranging from 50% to 100%) between each taxon (A-N) and the reference (*Nicotiana *top or *Zea *bottom). The height of the blue histogram topped by the horizontal black lines indicates the degree of similarity; similarity histograms are blue except where we have re-colored yellow the regions equivalent to *ycf15 *(top) and *ycf68 *(bottom) to highlight those regions. [The black horizontal lines without blue bars subtending them indicate short regions of similarity, basically SDRs. Red bars above the histogram indicate evolutionary conserved regions as determined in Mulan.] In interpreting the diagram, essentially the more blue (or yellow) in a region, the more similar are the two sequences. (Top) Comparisons, relevant to the conservation of *ycf15*, of six IGS regions from *Nicotiana tobaccum *were made to *Calycanthus floridus *(A), *Amborella trichopoda *(B), *Zea mays *(C), *Saccharum officinarum *(D), *Phalaenopsis aphrodite *(E), *Lotus japonicus *(F), *Acorus calamus *(G), *Arabidopsis thaliana *(H), *Spinacia oleracea *(I), *Oenothera elata *(J), *Eucalyptus globulus *(K), *Nymphaea alba *(L), *Nuphar advena *(M), and *Ranunculus macranthus *(N). (Bottom) Comparisons, relevant to the conservation of *ycf68*, of introns from *Zea mays *were made to those of *Ranunculus macranthus *(A), *Calycanthus floridus *(B), *Eucalyptus globulus *(C), *Lotus japonicus *(D), *Spinacia oleracea *(E), *Phalaenopsis aphrodite *(F), *Nuphar advena *(G), *Nymphaea alba *(H), *Arabidopsis thaliana *(I), *Nicotiana tobaccum *(J), *Oenothera elata *(K), *Amborella trichopoda *(L), *Acorus calamus *(M), and *Saccharum officinarum *(N).

To further investigate the properties of these two "*ycf*s" we applied two additional approaches: 1) graphing codon usage patterns, following Echols *et al*. [31], of these putative genes relative to that of known genes and non-coding regions; and 2) comparing the level of conservation in the *ycf15*- and *ycf68*-containing regions with other similar regions using Mulan [32]. Codon usage results were ambiguous (data not shown, see Methods). The Mulan results, though intuitive, were instructive (Figure 6). We compared the *ycf15*-containing intergenic spacer as well as five other similarly sized IGS (two from the IR regions and three from the LSC) to the homologous regions in *Nicotiana*, for each of 14 genomes (Figure 6 top). For *ycf68*, we did similar comparisons, using *Zea *as the reference taxon, of the *trnI*-GAU (*ycf68*-containing) intron, two other introns from the IR and three introns from the LSC (Figure 6 bottom). In both cases, but especially for the intron (*ycf68*) comparison, it can be seen that other IGS or intron sequences are as (or even more) conserved as the *ycf *regions and that non-coding sequences (introns or IGS) are much more conserved in the IR than in the LSC. Thus, it is seems likely that these regions (*ycf15 *and *ycf68*) are conserved, not because they serve some function, but simply because they are in the inverted repeat; they simply appear to code for a polypeptide chain of suggestive length due to chance and are conserved across large evolutionary distances because of the especially low rates of change within the IR [33].

### Codon Usage

We examined codon usage patterns in *Nuphar *and *Ranunculus *for the 79 protein-coding genes (i.e., not including the hypothetical genes, *ycf15 *and *ycf68*). We compared start codon usage in ten representative genomes being careful to compare homologous positions as much as possible. [This was straightforward among seed plants but sometimes problematic with *Huperzia*.] For five genes in each organism (with some but not all genes held in common), either ACG or GTG appear to be used as an alternative to ATG as the start codon, as is common for a variety of genes in the plastid genomes of seed plants (Table 4). In the pteridophytes, *Huperzia *(Table 4) and *Adiantum *[34], even more genes (10 and 26, respectively) use alternative start codons, and the pteridophyte repertoire includes GCG and ATT, in addition to ACG and GTG.

**Table 4:** Alternative start codon usage in selected land plant genomes.

Gene Species	*Huperzia*	*Pinus*	*Amborella*	*Nymphaea*	*Nuphar*	*Calycanthus*	*Ranaunculus*	*Arabidopsis*	*Nicotiana*	*Triticum*
*atpI*	ACG									
*cemA*			GTG	GTG	GTG	GTG				
*chlL*		GTG	-----	-----	-----	-----	-----	-----	-----	-----
*matK*	ACG									
*ndhB*		-----					ACG			
*ndhD*	ACG	-----	ACG		ACG	ACG	ACG		GTG	
*ndhG*	ATT	-----								
*petN*	GTG									
*psaJ*	GTG									
*psbL*			ACG	ACG	ACG	ACG			ACG	
*rpl2*			ACG				ACG			ACG
*rpl36*	ACG									
*rpoC2*	ACG									
*rps19*				GTG	GTG	GTG	GTG	GTG	GTG	GTG
*ycf1*	ACG									
*ycf2*	ATT						GTG			

Total Number	10	1	4	3	4	4	5	1	3	2

Pseudogenes and ORFs were not included. Dashes in a cell indicate that that gene is absent from that genome.

**Figure 7:**
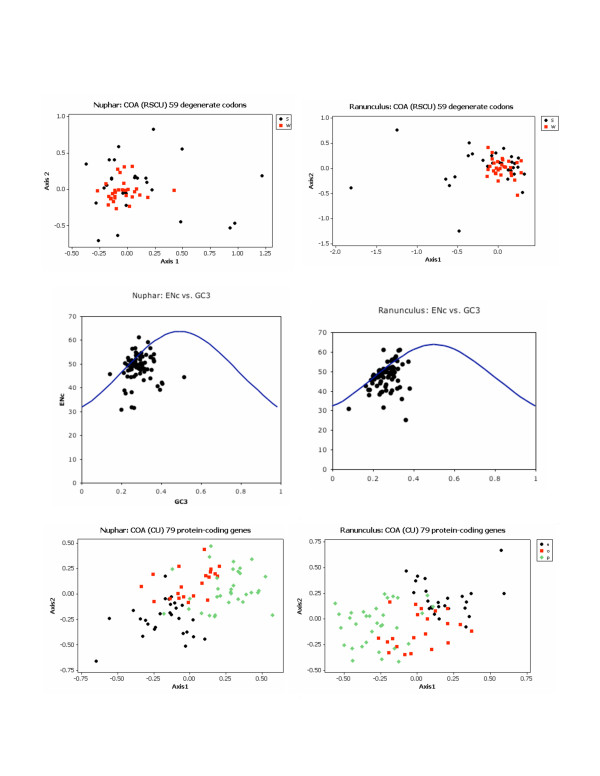
Graphical analyses of codon usage patterns. (top) Plots of the two most significant axes generated by the COA of RSCU values for *Nuphar *(top left) and *Ranunculus *(top right). Each point represents one of the 59 degenerate codons. The points are coded S (black circle) if the 3^rd ^position nucleotide is G or C, and W (red square) if the 3^rd ^position nucleotide is A or T. (middle) Plots of ENc (effective number of codons) by GC3 (the percentage G + C at the 3^rd ^position) for each of the 79 protein-coding genes in *Nuphar *(middle left) and *Ranunculus *(middle right). The line in each graph (middle left and right) indicates the relationship predicted if codon usage was determined solely by 3^rd ^position composition. (bottom) Plots of the two most significant axes generated by COA on CU (codon usage) for genes in *Nuphar *(bottom left) and *Ranunculus *(bottom right). Each gene is categorized as related to photosynthesis (green diamonds), gene expression (black circles) or other (red squares).

Overall codon usage in the *Nuphar *and *Ranunculus *genomes (Table 5) is generally similar to that reported from other genomes such as *Panax *[17], *Lotus *[35] and *Nicotiana *[36]. As in these and other genomes [5,6,36-38] where the genetic code is redundant, codons with a third position nucleotide of A or T(U) are used more frequently than those terminating in G or C (Table 5). The base composition at each of the three codon positions varies, with the first position having the lowest proportion of A+T and the third position the highest (Table 1). It has been suggested that codon usage patterns are driven by this composition bias [39,40]. However, when we apply methods to assess the impact of nucleotide composition on codon usage within *Nuphar *and *Ranunculus*, it appears that the A+T-richness of the third position is at most a partial influence on codon usage. We used CodonW [41] to calculate a variety of codon usage statistics that we then contrasted graphically (Figure 7). COrrespondence Analysis of codon usage (COA) was calculated based on codon usage as well as Relative Synonymous Codon Usage (RSCU). In each case the first two axes together explained only a modest amount of the variation (15–20%) and plotting each of the 56 degenerate codons on the first and second axes did not produce a pattern related to the A+T-richness of the third position in the codon sequence (Figure 7 top), except that codons ending in A or T are more tightly clustered than the codons ending in G or C. These results suggest that A+T-richness is not the most important factor in explaining variation in codon use. In addition we graphed each gene on the two axes ENc (the effective number of codons) and GC3 (the G+C percentage at the third position) (Figure 7 middle). If codon usage is random with respect to factors other than A+T-richness (i.e., GC content) of the third position, each gene is expected to fall on the prediction line for ENc based solely on its GC3 value [42,43]. Again our results reinforce the finding that factors other than nucleotide composition are operating in the plastid genome to select among synonymous codons across genes, in that most points do not fall on the prediction line. Other studies, for example Wall and Herbeck's study of codon bias in the plastid gene *rbcL *[44], also have found that codon usage patterns are not explained by G+C patterns. Finally, we calculated COA eigen values for each gene for the *Nuphar *or *Ranunculus *genome (based on codon usage, RSCU or amino acid usage, the results are comparable although more obvious for codon or amino acid usage). The eigen values for the primary axes are higher (explain a higher proportion of the variation) than those seen for codons (about 25% in the case of the codon usage analyses) and plotting each gene on the first two axes produces patterns (Figure 7 bottom) suggesting that different functional groups of genes have different codon usage and amino acid usage patterns as has been found in broader comparisons [6,36]. Overall, although A+T-richness of the third position is the most obvious pattern observable in plastome codon usage, other factors are important in determining codon usage patterns in particular genes (and perhaps genomes).

**Table 5:** Codon usage (codon frequency relative to each amino acid) for *Nuphar *(Nuad) and *Ranunculus *(Rama).

		Nuad	Rama			Nuad	Rama			Nuad	Rama			Nuad	Rama
F	TTT	**59.0**	**67.0**	S	TCT	**27.0**	**27.3**	Y	TAT	**77.6**	**80.1**	C	TGT	**66.2**	**76.7**
F	**TTC**	41.0	33.0	S	**TCC**	17.0	16.6	Y	**TAC**	22.4	19.9	C	**TGC**	33.8	23.3
L	**TTA**	**28.6**	**32.6**	S	**TCA**	21.2	19.4	*	TAA	**40.5**	**48.4**	*	TGA	30.4	25.3
L	**TTG**	21.3	21.0	S	TCG	8.1	10.1	*	TAG	29.1	26.4	W	**TGG**	100.0	100.0
L	CTT	19.7	21.4	P	CCT	**37.4**	**37.0 **	H	CAT	**75.4**	**75.0**	R	**CGT**	21.1	22.8
L	CTC	7.4	6.2	P	CCC	21.7	21.1	H	**CAC**	24.6	25.0	R	CGC	7.8	7.5
L	**CTA**	14.7	12.7	P	**CCA**	28.9	28.0	Q	**CAA**	**71.7**	**75.5**	R	CGA	21.5	22.5
L	CTG	8.3	6.0	P	CCG	12.1	13.8	Q	CAG	28.3	24.5	R	CGG	7.3	7.1
I	ATT	**47.1**	**51.9**	T	ACT	**39.7**	**37.8**	N	AAT	**77.4**	**77.4**	S	AGT	20.6	20.7
I	**ATC**	22.1	15.9	T	**ACC**	20.6	18.1	N	**AAC**	22.6	22.6	S	**AGC**	6.1	6.0
I	ATA	30.8	32.2	T	**ACA**	28.3	31.6	K	**AAA**	**71.3**	**75.2**	R	**AGA**	**30.1**	**29.0**
M	**ATG**	100.0	100.0	T	ACG	11.3	12.5	K	AAG	28.7	24.8	R	AGG	12.3	11.1
V	GTT	35.1	37.9	A	GCT	**45.9**	**41.5**	D	GAT	**78.7**	**78.7**	G	GGT	34.5	34.2
V	**GTC**	12.7	10.7	A	GCC	15.5	16.1	D	**GAC**	21.3	21.3	G	**GGC**	10.3	9.2
V	**GTA**	**36.1**	**38.5 **	A	**GCA**	27.4	29.0	E	**GAA**	**72.8**	**71.9**	G	**GGA**	**38.0**	**38.6**
V	GTG	16.1	12.9	A	GCG	11.2	13.4	E	GAG	27.2	28.1	G	GGG	17.1	17.9

Codons shown in bold complement the anticodons of the tRNAs encoded in the chloroplast genome. Frequencies shown in bold indicate the most common codon (where synonymous codons exist for that amino acid or termination).

### Comparisons of Nuphar and Nymphaea

Between the *Nuphar *and *Nymphaea *chloroplast genomes, we compared each individual gene, intron or intergenic spacer (IGS) region and calculated percent similarity (Table 6, Figure 1). For summary calculations, only one copy of each region in the IR was included and identities for multiple introns within a single gene were calculated separately. More than two-thirds of *Nuphar *regions match the homologous region of *Nymphaea *at a similarity level of 95% or higher; only 3% of the genomes fell below a threshold of 70% identity. As expected, coding regions are more highly conserved than IGS on average, although not in all cases. All four genes for rRNAs are 100% identical and those for tRNAs have at least 95% identity. Three protein-coding genes are 90–94% identical and all others are at least 95% identical. In some cases IGS and introns are more similar than coding regions – 60% of introns and 41% of IGS are at least 95% identical in sequence between the two genomes. Interestingly, the distribution patterns of the numbers of IGS and the numbers of introns in the different percent identity categories appeared quite similar, suggesting that similar forces may impact both types of non-coding sequences.

**Table 6:** Number of *Nuphar *plastome regions attaining different sequence identities relative to homologous *Nymphaea *plastome regions.

**Region**	**99–100%**	**95–98%**	**90–94%**	**80–89%**	**70–79%**	**0–69%**	**Total**
**Protein**	17	63	3	0	0	0	83
**Intergenic**	16	29	27	23	8	7	110
**Introns**	4	8	5	2	1	0	20
**rRNA**	4	0	0	0	0	0	4
**tRNA**	19	11	0	0	0	0	30

**Total**	60	111	35	25	9	7	247

Detailed percent identity comparisons are rarely reported for individual regions of entire chloroplast genomes. One study compared sugar cane with rice, maize and wheat and reported comparisons as one of three categories: 0–30%, 31–79% and 80–100% [45]. As one might expect, most of the regions fell into the latter category. Timme *et al*. [46] compared sequence divergence in both coding and non-coding regions between two completely sequenced chloroplast genomes from representatives of two of the subfamilies of Asteraceae. Their results showed that intergenic spacer regions were nearly two times as divergent as introns, and that the 10 most divergent coding sequences represent several different functional groups, including photosynthetic genes, ribosomal proteins, and *ndh *genes. Another study compared divergence within functional groups across four different species in three genera of Solanaceae and found that RNA and photosynthesis genes are the most conserved [47], consistent with the results from a second Solanceae comparison based on individual coding regions [48]. Kim and Lee [17], in their comparison of coding regions (omitting the tRNA genes) from 16 fully sequenced vascular plant chloroplast genomes, found the four rRNA genes to be the least divergent, followed *by psbA, psbD, rps12, psbE, psbL *and *petB*. The most conserved coding regions in *Nuphar-Nymphaea *are similar in that the four rRNA genes, *rps12, psbL *and *petB *are also among the most conservative genes but differ in including *petN, psbM*, and *rpl23 *among the 10 most conserved coding regions.

We also examined indels (insertions and deletions) between *Nuphar *and *Nymphaea *which, of course, are much more likely to occur in IGS than in coding regions. In our study, 88.6% of insertions and 89.9% of deletions occurred in IGS (data not shown), comparable to results from a similar comparison between sugarcane and maize where 84.9% of insertions and 74.2% of deletions occurred in IGS [45]. In another study, the comparison of two varieties of rice (*Oryza sativa*) found 110 indels between those two plastid genomes [49], whereas we found almost four times as many (413), but, of course, our taxa are less closely related. However a study comparing *Atropa belladonna *to *Nicotiana tabaccum*, a comparison perhaps more comparable to ours, found 65 insertions and 60 deletions equal to or larger than five bp in intergenic regions and introns [50], whereas we found 163 insertions and 206 deletions in *Nuphar *relative to *Nymphaea*. Together the combined lengths of the indels account for 0.08% of the total genome length of *Atropa *and 0.08% of the genome length in the rice comparison, but 0.23% of the genomic length in the *Nuphar-Nymphaea *comparison.

Only recently have plastome sequences been available for closely related taxa, allowing for detailed comparisons [46-48]. These comparisons suggest that non-coding sequences within the genome evolve more rapidly in terms of both substitution and indel mutations, although this is not universally so (some non-coding sequences are quite highly conserved). Comparisons across studies show that some regions are consistently slow to evolve and others commonly evolve at a higher rate but at least minor differences are seen across these studies. Before general patterns and processes can be identified more genomic data allowing for such comparisons will need to become available.

### Repeat Analysis – Simple Sequence Repeats (SSRs)

We screened for perfect SSRs in *Nuphar*, *Ranunculus *and 24 other chloroplast genomes (Table 7). We report the number of mononucleotide repeats ≥ 8 nt, dinucleotide repeats ≥ 8 nt (i.e., four repeat units), and trinucleotide repeats ≥ 9 nt (i.e., three repeat units); hereafter referred to collectively as the 8,8,9 SSRs. We also report the number of longer repeats ≥ 10 nt/repeats for mononucleotide runs, ≥ 10 nt (or five copies of the repeat unit) for dinucleotide repeats, and ≥ 12 nt (i.e., four copies of the repeat unit) for trinucleotide repeats; referred to collectively as the 10,10,12 SSRs. Of course, any particular threshold (e.g., 8,8,9) is rather arbitrary and no consensus has developed on what nucleotide length or repeat unit number is significant [51]. However, it has been suggested that SSRs of length 8 nt or more (regardless of repeat motif) are prone to slip-strand mispairing (SSM, thought to be the primary mutational mechanism to affect SSRs), whereas those of lesser length are not [52]. Elsewhere the critical threshold is estimated at 7–10 bp [53]. Other workers have chosen similar thresholds, of 8 or 10 nt, in their reports [e.g., [54]]. The number of 8,8,9 SSRs vary from 152 in *Pinus thunbergii *to 393 in *Lotus *and comprise between 1 and 2.5% of the chloroplast genome. The number of 10,10,12 SSRs range from 16 in one of the rice genomes to 113 in *Medicago*.

**Table 7:** Number and maximum length of SSRs present in twenty-six land plant chloroplast genomes.

			8,8,9 SSRs (number)				10, 10,12 SSRs (number)						
						
Taxon	Accession Number	Genome size	mono	di	tri	**total**	mono	di	tri	**total**	max mono (units)	max di (units)	max tri (units)
*Huperzia lucidula*	NC_006861	154,373	147	34	80	**261**	25	10	7	**42**	15	9	10
*Psilotum nudum*	NC_003386	138,829	111	35	58	**204**	28	12	3	**43**	17	6	5
*Adiantum capillus-veneris*	NC_004766	150,568	146	32	39	**217**	94	4	2	**100**	19	6	4
*Pinus thunbergii*	NC_001631	119,707	74	36	42	**152**	20	5	0	**25**	17	7	3
*Pinus koraiensis*	NC_004677	116,866	77	38	39	**154**	36	6	0	**42**	23	9	3
*Oryza nivara*	NC_005973	134,494	91	20	42	**153**	10	4	3	**17**	13	5	4
*Oryza sativa indica 93-11*	NC_008155	134,496	94	20	42	**156**	9	4	3	**16**	16	5	4
*O. sativa japonica Nipponbare*	AY522330	134,551	94	20	42	**156**	11	4	3	**18**	17	5	4
*O. sativa japonica PA64S*	AY522331	134,551	94	20	42	**156**	11	4	3	**18**	17	5	4
*Saccharum officinarum*	NC_006084	141,182	128	26	47	**201**	32	5	1	**38**	15	5	4
*Zea mays*	NC_001666	140,384	121	29	52	**202**	34	6	2	**42**	18	6	5
*Triticum aestivum*	NC_002762	134,545	116	33	43	**192**	24	8	3	**35**	15	6	5
*Nuphar advena*	NC_008788	160,866	71	65	84	**220**	19	23	10	**52**	16	11	5
*Nymphaea alba*	NC_006050	159,939	63	60	73	**196**	15	4	4	**23**	16	5	4
*Amborella trichopoda*	NC_005086	162,686	101	47	57	**205**	35	8	6	**49**	15	9	4
*Calycanthus fertilis*	NC_004993	153,337	105	35	93	**233**	14	8	4	**26**	13	8	4
*Ranunculus macranthus*	NC_008796	155,158	146	60	55	**261**	28	9	3	**40**	16	8	5
*Arabidopsis thaliana*	NC_000932	154,478	234	83	61	**378**	69	18	6	**93**	17	8	5
*Oenothera elata*	NC_002693	163,935	155	48	68	**271**	56	6	8	**70**	24	6	4
*Panax ginseng*	NC_006290	156,318	92	39	60	**191**	18	5	3	**26**	13	7	4
*Nicotiana tabacum*	NC_001879	155,939	116	41	73	**230**	38	7	5	**50**	17	5	4
*Atropa belladonna*	NC_004561	156,687	116	46	74	**236**	39	10	2	**51**	17	6	4
*Spinacia oleracea*	NC_002202	150,725	146	55	64	**265**	40	9	4	**53**	12	7	4
*Epifagus virginiana*	NC_001568	70,028	106	36	50	**192**	25	16	4	**45**	15	10	5
*Lotus japonicus*	NC_002694	150,519	236	80	77	**393**	76	27	6	**109**	16	11	5
*Medicago truncatula*	NC_003119	124,033	190	63	93	**346**	76	28	9	**113**	18	7	6

**Figure 8:**
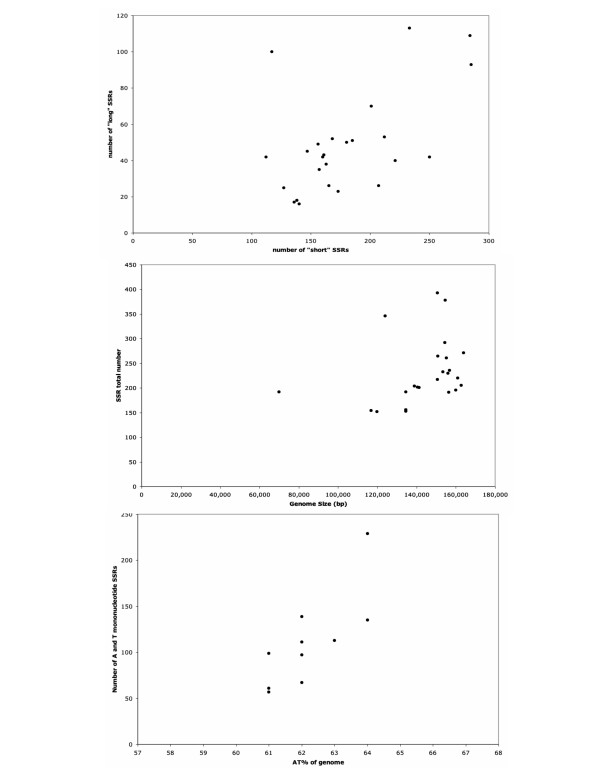
Scatter plots showing relationships between aspects of SSR frequency and other characteristics. (top) The relationship of "short" SSRs and "long" SSRs. "Long" SSRs are the 10,10.12 repeats. "Short" SSRs are the 8,8,9 repeats with the 10,10,12 repeats excluded. These are shown for the 24 taxa in Table 7. (middle) The relationship between total SSR number and genome size (in nucleotides) for the 24 taxa. (bottom) The relationship of A+T-richness (the overall A+T percentage of the genome) and the frequency of A and T mononucleotide repeats for the 10 taxa involved in the more detailed comparison. No other SSR category showed a relationship to any aspect of nucleotide composition.

We calculated, based on the data presented in Table 7, Spearman Rank Correlation statistics [using [55]] to look for relationships between the number of short and long SSRs and between genome size and the total number of SSRs (Figure 8). If some genomes are generally more likely to contain SSRs (due to differences in mutational biases or selection pressures or other factors) and a common mechanism (or suite of mechanisms) controlled the likelihood of SSR presence, then a correlation between short and long SSRs would be predicted. Alternatively if some genomes were predisposed to long SSRs whereas others were less likely for SSRs to attain greater length, then no correlation might be seen or even a negative correlation might be observed. Genome size and total number of SSRs should be correlated if SSRs occur randomly. Both these comparisons showed a small but significant positive relationship. Genomes with a higher number of "short" SSRs (from the first to the second threshold) were more likely to have a higher number of "long" SSRs (at or above the second threshold) – Figure 8 (top), r_s _= 0.534, p = 0.009. Larger genomes were more likely to contain more SSRs than smaller genomes – Figure 8 (middle), r_s _= 0.542, p = 0.008 (r_s _= 0.524, p = 0.012 with *Epifagus *excluded). Thus we can infer that the larger the genome the more SSRs are to be expected and that "long" and "short" SSRs most likely are simply points on a continuum evolving under similar mechanisms. However these two factors explain only a portion of the variance in SSR number.

For a subset of the chloroplast genomes, we conducted a more detailed comparison of the SSRs (data shown in additional file 1), including all of the then available basal angiosperms and basal eudicots, plus a pteridophyte, a conifer, two derived eudicots and one monocot. In these comparisons we characterized repeats based on nucleotide composition in addition to length and number. We then compared (data not shown) the predicted and observed number of repeats of various types and the predicted and observed ratios between repeats of various compositions. For all types of repeats, of all lengths and all compositions, in all genomes, fewer SSRs were observed than were predicted. In many cases the difference between the number of SSRs predicted and the number observed was more than an order of magnitude. For mononucleotide repeats in most angiosperms, the ratio of A or T (W) containing runs to G or C (S) containing runs was not significantly different from the ratio predicted from the genomic base composition. However, in the two Nymphaeaceae (*Nuphar *and *Nymphaea*), *Pinus *and *Huperzia*, the observed ratio of W to S mononucleotide SSRs was significantly different from the predicted ratio (Chi-Square test, p < 0.001). Interestingly the observed ratio was skewed in favor of S (G and C) containing repeats. When predicted ratios of dinucleotide repeats were calculated based on the overall nucleotide composition, all genomes showed a significantly biased ratio (Chi-square test, p < 0.001). In this case more WS SSRs were observed than expected and fewer WW SSRs. When the dinucleotide calculations were repeated based on the genomic dinucleotide frequencies, the deviation of the observed from the expected was less and for *Arabidopsis *and *Triticum *was not significant. Again where there is a deviation from expected, the WS category is elevated and the WW category is reduced in the observed. Finally, in calculations related to trinucleotide SSR compositions and based on overall trinucleotide composition, all but *Arabidopsis *and *Triticum *showed biased compositional ratios. Bias was due primarily to fewer WWW SSRs than expected. Thus, surprisingly, whenever the observed compositional ratio of SSRs was different from expectation (the majority of cases), the deviation was due to a deficiency of W (A and T) containing repeats.

Other (non-plastid) genomes that are A+T rich have been found to exhibit a strong overrepresentation of A or T mononucleotide SSRs, e.g., *Caenorhabditis elegans *[56] and *Plasmodium falciparum *[53] and various explanations for this have been offered. Because A-T base pairs are held together by two hydrogen bonds rather than three, A-T containing repeats are easier to denature and therefore perhaps more prone to slip-strand mispairing (SSM). Other explanations include mutational bias (i.e., G-C to A-T mutations being more likely than the reverse [56]), involvement of A-T runs in gene regulation [53], and regions being more mutagenic due to A-T runs (this increased mutability being selected for in regions where higher mutations rates would be advantageous [56,57]). Although these proposals seem reasonable, SSRs (and specifically A and T containing SSRs) are underrepresented in the plastid genomes in contrast also to the work of Dieringer and Schlotterer [58] who found SSRs overrepresented when comparing nine nuclear genomes. They predicted that the more biased from equal nucleotide composition the genome the greater the density of microsatellites (either mono- or di- nucleotide SSRs in their case). For the genomes considered in our detailed comparison, we tested for correlation between various aspects of nucleotide composition and SSR frequency. Only A and T mononucleotide repeat density correlated with genomic nucleotide composition (Figure 8 bottom, r_s _= 0.81, p = 0.015). So this correlation can be seen even when the W repeats are not over represented. G and C mononucleotide repeat density did not correlate with nucleotide composition and di- and tri-nucleotide repeat densities correlated neither with genomic frequencies nor with di- or tri-nucleotide frequencies calculated over the entire genome (data not shown). We note that GpC and CpG based (SS) repeats are completely absent from all the plastid genomes examined. This may be the result of selection against these motifs in addition to simple nucleotide composition (these would be predicted to be very rare; about one SS repeat of length 8 nt per genome on average) as SS-based dinucleotide repeats are absent or strongly underrepresented in other genomes as well [51,58,59]. Presumably some mechanism (mutational or selectional) in the chloroplast genome is acting on the plastid genomes such that many fewer SSRs are observed than would be expected and that mechanism is acting more strongly on W-containing repeats than S-containing ones.

In an alternative attempt to clarify underlying patterns and mechanisms of SSR evolution, we compared the 10,10,12 SSRs between *Nuphar *and *Nymphaea *to determine how many of the SSRs were shared (determined by identity of flanking sequence and repeat position as well as repeat motif) between these two genomes from relatively closely related plants (additional file 2). The *Nuphar *genome contains more than twice as many 10,10,12 SSRs as the *Nymphaea *genome – 52 in *Nuphar*, 23 in *Nymphaea*. Sixty-six different 10,10,12 SSRs occur in the combined set. The vast majority, but not all, of the SSRs occur in non-coding DNA (55 in IGS, six in introns, and five in coding regions). The majority (50 of 66) of these repeats are shared between the two genomes; most are simply lower than the reporting threshold in one genome or the other. Of the 50 shared SSRs, only seven are the same length in both genomes. Where the shared SSRs are of different lengths, in 18 cases length differences were due to SSM, in 14 cases due to a nucleotide substitution, in four cases due to an indel (a length mutation other than a change in repeat unit number) and in eight cases due to a combination of mechanisms. Of the 16 SSRs not shared, 14 were found only in *Nuphar *and only two in *Nymphaea*. Thus, it appears that *Nuphar *is more likely to have these SSRs and for the SSRs to be longer when they occur; in 30 of the 43 cases where shared repeats differ in length the *Nuphar *SSR is longer.

It is thought that SSRs begin as random runs of nucleotides [51,60]. Any bias in mutation patterns or nucleotide composition would make certain runs more likely. Then, once present in a location, the repeat would grow via SSM [51,57,58,60]. Longer SSRs lead to more stable heteroduplex intermediates, making SSM more likely [57]. However longer SSRs also have higher mutations rates [51]. One model of SSR evolution posits that the distribution of repeat lengths in a genome represents an equilibrium between SSM and point mutation [51]. In the *Nuphar-Nymphaea *comparison SSM and point mutation occur with about equal frequency, consistent with this hypothesis. In terms of phylogenetic utility of SSR variation, the *Nuphar-Nymphaea *comparison suggests that individual SSRs are stable at least over relatively short periods of evolutionary time and that they do commonly vary in repeat number. However the small size of most repeats probably limits their utility and more needs to be known about the specifics of SSR evolution before any phylogenetic utility can be fully realized. Understanding more about the processes of SSR evolution will also help us investigate possible selective or functional roles for these motifs.

### Repeat Analysis – Small Dispersed Repeats (SDR)

**Figure 9:**
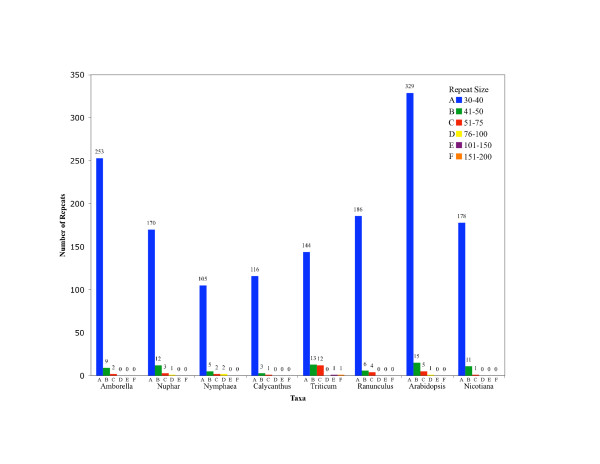
The number of SDRs of different length classes found in eight different angiosperm plastid genomes. The majority of repeats are 40 nt or less in length, but some genomes so have repeats that are longer. *Triticum*, the only genome to have repeats over 100 nt in length, is also the only genome to exhibit inversions changing aspects of gene order from the angiosperm consensus order exhibited by *Nicotiana *(and the other genomes included.)

We also searched for SDRs in the plastomes of representative angiosperms. These repeats are based on a more complex motif and are longer than SSRs. Our SDR analysis, within each of the eight genomes, identified 114–350 direct and inverted repeats 30 bp or longer with a sequence identity of at least 80% (Figure 9). The number of repeats was lowest in *Nymphaea *(114) and highest in *Arabidopsis *(350). In most cases, the number of direct repeats (62–208) was substantially higher than the number of inverted repeats (32–142). The vast majority (84–97%) of the repeats were only 30–40 bp in length and the longest repeat was 193 bp in *Triticum*.

Blast comparisons of the repeats identified in each of the genomes were performed against all other genomes to locate shared repeats with an e-value of 2. Although these comparisons were performed using the repeats in each of the eight genomes as the reference, we only present the results (Table 8) using *Nymphaea *as the reference genome because it had the fewest number of repeats and thus would contain any repeats shared throughout all these genomes. Overall, the analyses identified 83 groups of shared repeats among these eight angiosperm chloroplast genomes, ranging in length from 30 to 49 bp. The majority of the shared repeats were located within intergenic spacer regions and introns. However repeats were sometimes found within genes and some cases represented inter-tRNA similarities within different families of tRNA genes. The largest shared repeat (49 bp) represents shared sequence between *psaA *and *psaB*. In general, the longer shared SDRs (36 – 49 bp) tend to have lower A+T-richness (at the level of the genome or lower). However, most (68 of the 83) of the shared SDRs are short, between 30 and 32 nt in length. This class of SDRs shows the widest range in A+T-richness (29% to 97%) but most exhibit A+T-richness of greater than 50%. Most shared SDRs are present in only a small number of copies. Only two repeats occur more than four times in the genomes (both are found in introns and IGS and exhibit A+T richness less than that of the genomes overall); one is 42 nt long (51% AT) and the other, occurring about 10 times, is 40 nt long (54% AT). Overall, no trends were detected between repeat location, number, length, and A+T-richness.

**Table 8:** Shared repeats among eight angiosperm chloroplast genomes.

**Repeat Size (AT%)**	***Nymphaea *(reference genome)**	***Amborella***	***Nuphar***	***Calycanthus***	***Triticum***	***Ranunculus***	***Arabidopsis***	***Nicotiana***	**Location**
49(64)	2	2	2	2	2	2	2	2	*psaA*, *psaB*
43(61)	4	4	4	4	0	4	2	2	*ycf2*
42(51)	5	6	7	7	5	5	6	7	intron *ycf3*, intron *rpoC1*, IGS *psbH*:*petB*, IGS *petD*:*petB*, IGS *rpl16*:*rps3*, IGS *trnV*:*rps12 *3'
40(54)	11	10	11	11	11	7	9	10	intron *ycf3*, intron *ndhB*, IGS *trnV*:*rps12 *3', IGS *rpl16*:*rps3*, IGS *psbH*:*petB*, IGS *rps12 *3':*rps7*
37(39)	2	2	2	2	1	2	2	2	IGS *trnS*:*trnR*
36(51)	3	2	3	2	2	3	2	3	*trnV*, *trnA*
35(75)	2	0	1	0	0	0	0	0	IGS *rps12 *5':*clpP*
35(47)	3	3	3	3	3	3	3	3	*trnS*
35(100)	3	2	4	1	0	2	4	1	intron *trnL*, IGS *ndhC*:*trnV*
34(91)	2	2	2	2	2	1	2	2	*trnfM*, *trnP*
34(86)	2	0	2	0	0	0	0	0	IGS *atpH*:*atpI*, IGS *trnS*:*trnR*
34(54)	4	1	1	0	1	0	4	0	intron *trnL*, IGS *trnE*:*trnT*
33(59)	2	3	2	2	3	2	3	2	intron *atpF*, IGS *trnS*:*trnR*
33(85)	2	2	1	1	0	1	1	2	intron *clpP*, *rpoC2*
33(47)	4	4	4	2	4	4	4	4	*trnI*, *trnN*
32(79)	2	0	1	1	0	0	0	0	IGS *psbA*:*matK*
32(94)	2	0	1	0	0	0	0	0	IGS *trnP*:*psaJ*
32(58)	3	3	3	3	4	3	3	3	*trnT*, *trnM*
32(64)	2	2	2	2	1	2	0	2	*psaA*, *psaB*
32(97)	3	1	0	1	1	1	4	1	intron *trnL*, IGS *ndhC*:*trnV*
32(69)	2	0	2	1	1	1	1	2	intron *ndhA*, *rpoA*
31(66)	4	2	4	4	0	6	2	4	*ycf2*
31(66)	2	2	2	2	2	2	2	2	IGS *trnS*:*trnR*, *trnG*
31(56)	2	1	2	2	1	2	2	2	Intron *ndhA*, IGS *rpl16*:*rps3*
31(59)	2	2	2	2	2	2	2	2	*trnT*
31(53)	2	1	2	1	3	1	1	3	intron *ycf3*, intron *ndhA*
31(66)	4	4	4	4	0	2	4	4	*ycf2*
31(75)	2	2	2	2	0	2	1	2	intron *ndhB*
31(88)	2	0	1	0	0	0	0	0	IGS *rps16*:*trnQ*, *cemA*
31(84)	2	1	2	1	0	1	0	0	intron *ndhB*, IGS *trnT*:*psbD*
31(34)	2	2	2	1	0	2	1	1	*psaB*, *psaA*
31(56)	2	2	2	1	1	1	1	1	*psaB*, *psaA*
31(59)	2	0	2	0	0	0	0	0	IGS *rps12 *5':*clpP*
31(75)	2	0	2	0	0	0	0	0	intron *clpP*, intron *rps16*
31(84)	2	0	1	0	0	0	0	0	IGS *rps15*:*trnN*
30(77)	2	1	0	0	0	0	0	0	intron *ycf3*
30(52)	2	2	2	2	2	2	2	2	*trnS*
30(71)	3	3	3	3	4	4	3	3	intron *ndhB*, IGS *trnS*:*trnR*
30(52)	3	3	3	3	3	3	3	4	intron *ycf3*, IGS *trnV*:*rps12 *3'
30(55)	2	1	2	0	0	0	0	0	IGS *petA*:*psbJ*
30(42)	4	2	4	2	2	2	2	2	IGS *trnV*:*rps12 *3', *rrn23*
30(39)	4	2	4	2	2	2	2	2	IGS *trnV*:*rps12 *3', *rrn23*
30(68)	2	2	2	1	2	2	2	1	intron *ndhA*, IGS *trnS*:*trnR*
30(84)	3	0	2	0	0	0	0	0	*rpl23*
30(68)	4	4	4	4	0	4	6	6	*ycf2*
30(74)	4	4	4	6	0	2	8	4	*ycf2*
30(55)	2	1	2	1	0	0	0	0	IGS *trnH*:*psbA*, *rpoB*
30(74)	2	0	2	0	0	0	0	0	IGS *psbK*:*psbI*, IGS *psbF*:*psbE*
30(97)	2	0	1	0	0	0	0	0	IGS *atpH*:*atpI*
30(90)	2	1	1	0	0	0	1	0	IGS *atpH*:*atpI*, IGS *psaI*:*ycf4*
30(68)	2	1	2	1	0	1	1	0	IGS *accD*:*psaI*, *rpoB*
30(74)	2	0	2	0	0	0	0	0	IGS *ycf6*:*psbM*, IGS *psaI*:*ycf4*
30(68)	3	3	3	3	0	3	3	3	IGS *psbD*:*psbC*, IGS *rpl14*:*rpl16*, *ycf2*
30(71)	2	2	2	2	2	2	2	1	*psaB*, *psaA*
30(55)	3	1	3	0	2	3	1	1	intron *ycf3*, IGS *trnV*:*rps12 *3'
30(65)	2	0	1	0	0	0	0	0	IGS *trnS*:*rps4*, IGS *rps15*:*trnN*
30(74)	2	2	2	2	1	1	2	1	*rps4*, *ndhC*
30(65)	2	1	2	2	0	1	0	2	*ndhK*, *ndhD*
30(61)	3	1	3	1	1	1	1	1	intron *trnV*, IGS *trnV*:*rps12 *3'
30(58)	2	2	1	0	0	1	1	1	*atpE*, *ndhD*
30(74)	2	1	1	1	1	1	1	0	IGS *petB*:*petD*, IGS *rps15*:*trnN*
30(71)	4	2	4	1	2	2	4	4	*rpl2*, *ycf2*
30(52)	4	2	4	1	2	2	2	2	*rpl2*, *ycf2*
30(29)	2	2	2	0	0	0	0	0	IGS *trnI*:*rrn16*

Repeats size is for *Nymphaea*, which was used the reference genome in Blast comparisons. Numbers in parentheses are percentage AT in each repeat. Numbers listed under each taxon indicate the number of copies of repeat shared with *Nymphaea*. The location indicates position of shared repeats. IGS = intergenic spacer.

Repeated sequences are generally considered to be uncommon in chloroplast genomes with the notable exception of the large IR present in most land plants [7]. Dispersed repeats are found more commonly in genomes that have experienced changes in genome organization [16,61,62], especially in highly rearranged algal genomes [63,64]. A correlation between the number of repeats and the extent of gene order change has been suggested for algal genomes [64]. However, comparisons of completely sequenced chloroplast genomes of the angiosperm families Fabaceae [65], Solanaceae [47], and Asteraceae [46] have revealed the presence of numerous small repeats scattered throughout these genomes even though these genomes have few if any rearrangements. In each of these families assessed by others, most of the repeats are 20 – 40 bp in length and they are located mostly in intergenic spacer regions and introns, although several are located in the protein-coding genes *psaA*, *psaB*, and *ycf2*. Our examination of repeats here in eight angiosperm chloroplast genomes, representing a wider phylogenetic diversity than that of earlier studies, identified numerous repeats in each genome of a nature and pattern similar to those reported by others based on narrower comparisons (Figure 9). Again here, some of the shared SDR repeats, such as those located in tRNA genes and those shared between *psaA *and *psaB*, result from conserved sequence similarity of related genes. The vast majority of the plastome SDRs are restricted to intergenic spacer regions and introns and are small in size. Earlier work suggesting that larger repeats (of a size detectable via Southern Hybridization) are rare in unrearranged plastid genomes is supported. However, the pattern that seems to be emerging from all of these analyses is that small SDRs in angiosperm genomes are quite common and they tend to be located in the same regions. The role of these conserved repeats is not known but given that many of them are shared broadly and are located in the same regions suggests that some may be functional.

## Conclusion

As additional chloroplast genomes from less-derived angiosperm taxa are characterized we obtain a clearer picture of the ancestral plastid genome organization for angiosperms. In large part these additional genomes (reported here and by others) confirm that the *Nicotiana *plastome is reasonably inferred to represent the ancestral angiosperm for gene content and organization, although perhaps not for exact IR boundaries. With notable exceptions, these features, seen in *Nicotiana*, are remarkably conserved in most angiosperm lineages. The *Nuphar advena *and *Ranuculus macranthus *chloroplast genome sequences add to this growing body of data for reconstructing the evolution of plastid genomes. The *Nuphar *genome, in addition, provided the opportunity for comparison with the relatively closely related *Nymphaea alba *plastome sequence. This comparison confirmed views on the conservative nature of the genome, with even some IGS regions showing very high levels of nucleotide similarity. The *Nuphar-Nymphaea *comparison also supported the view that SSR frequencies represent a balance between two mutation types: SSM and substitutions. Detailed comparisons among these and other genomes reveal many differences and unexplained conservation of features that both remain to be understood. However, we are able to suggest that the widely conserved sequences designated *ycf15 *and *ycf68 *are not protein-coding genes.

As has been noted many times elsewhere, chloroplast genomes are biased towards A and T nucleotides, i.e., are "A+T-rich", except for the RNA genes. Howe *et al*. [66] suggest that the A+T-richness of plastid genomes is the result of endosymbiosis (or at least enhanced due to endosymbiosis). They argue that there might be a selective advantage for a particular protein-coding gene to be either A+T-rich or G+C-rich and that each class of genes can be maintained through compartmentalization in the different genomes [66]. However, many other genomes, prokaryotic or eukaryotic, are as A+T-rich as (or even more biased than) plastid genomes [31,53]. We suggest that the plastome A+T bias is relatively modest and results from a slight mutation and/or error checking bias of the plastid DNA polymerases or perhaps some selection for A and T in otherwise neutral positions to increase ease of denaturation during replication or transcription. In any event, a bias can be seen in overall composition, in the composition of the 3^rd ^position of codons, and in which SSR motifs are most abundant, among other aspects of the genomic sequence. However on closer inspection A+T richness does not correlate with variation in codon usage or details of SSR abundance. We speculate that the interesting patterns are those that fail to track patterns of A+T-richness; for example, repeat sequences in non-coding regions enriched for G and C are the ones more likely to be functional and understanding the components of codon bias that is not related to A+T-richness is more likely to be significant.

## Methods

### DNA Sources

Leaf material of *Nuphar advena *(Aiton) W. T. Aiton was obtained from a cultivated plant (vegetatively propagated from wild material from Lake Moshanon, Center County, PA) in the Biology Greenhouse at Pennsylvania State University. A voucher made from this same plant (collection Claude W. dePamphilis 2001.301) was deposited at PAC.

*Ranunculus macranthus *Scheele leaf material was collected from a wild population in Austin, TX. Leaves from multiple individual plants were combined to provide enough material for the cpDNA isolations. A voucher was made from a plant from this population and deposited in TEX.

### Isolation Methods

*Nuphar advena *chloroplast DNA was prepared by the sucrose gradient method [67]. However, the sucrose-gradient method did not yield pure enough cpDNA from *Ranunculus macranthus*. Therefore, for *Ranunculus macranthus *the NaCl method [68] was used and yielded concentrated, sufficiently pure cpDNA and was used as the sequencing template. Our isolation methods are described in detail elsewhere [69].

### Shotgun Sequencing and Finishing

Details of our methodology for producing finished genomic sequences from the sequencing template (cpDNA in this case) are provided in Jansen et al [69]. We will provide an overview of our approach here.

Draft genomic sequence was prepared from the cpDNA preparations at the DOE Joint Genome Institute. To do so, the DNA was sheared by passage through a narrow aperture and then fragments averaging 3 kb were selected from an agarose gel and cloned into plasmids, which were then used to transform *E. coli*. Clones were randomly selected from these libraries and placed into 384-well plates and templates amplified for sequencing using rolling circle amplification. When sequenced, forward and reverse plasmid sequencing primers produced 500–750 bp of sequence data (reads) from each end of the inserts. The individual reads were then processed and assembled into contigs using Phred and Phrap [70,71]. Four or five plates (generating 768 reads each) provide 8–10× coverage if the library is 60–80% cpDNA.

In the case of *Nuphar*, one library was constructed. The sequencing reads from five plates assembled into a draft genome composed of a single contig. In the case of *Ranunculus*, three libraries were constructed and fourteen plates of sequence data were generated. These data assembled into a draft genome composed of a single contig. The draft sequences were then assessed visually using Consed [72] to determine the level of quality of each nucleotide. Each nucleotide in each read was assigned a quality score using Phred [70]. Our minimum criterion was two reads with a quality score of ≥ 20 for each position. At the vast majority of positions this criterion was greatly exceeded, but occasionally areas of low coverage occurred and there were some instances where the minimum criterion was not met. In those cases, we designed primers to flank the regions of these "quality gaps", PCR amplified a product that contained the questionable nucleotide or nucleotides, and sequenced the PCR product until the criterion was met. Five regions of low quality were confirmed in this manner in *Nuphar *and two in *Ranunculus*.

We also confirmed the extent of the IR with a PCR and sequencing strategy. In the shotgun sequencing approach, the two copies of the IR are not sequenced separately. Like the remainder of the genome, the sequence for the IR region is built up via the overlap and assembly of the 500–700 bp sequencing reads. Sequences derived from templates representing both copies of the IR assemble together. The IR can be recognized generally in Consed as the depth of sequencing reads doubles in that region and the boundaries can be inferred where the two reads from a single clone assemble far from each other. However to precisely define the boundaries we designed primers to amplify across each of the four IR-single copy junctions, sequenced the PCR products and compared those sequences to one another and to the draft genome. In this manner we were able to confirm the precise location of the IR-LSC and IR-SSC boundaries.

### Annotation and related studies

We used DOGMA [73] as our primary tool for annotating these two genomes. DOGMA uses BLASTX [74] to compare the genomic sequence against a custom database of genes constructed using corrected annotations of 17 completely sequenced chloroplast genomes. This produces a draft annotation that is then inspected using DOGMA's tools for accurate assessment of the start and codon of each gene and any contained exon-intron boundaries. Because of the limitations of BLAST searches, small exons (6–9 nucleotides) that occur in three chloroplast genes cannot be found by DOGMA or by using other versions of Blast searches, so these were located manually. Putative gene and exon boundaries are determined by detailed comparison with other annotated genomes and individual gene sequences; no expression or protein studies were conducted to confirm the assignments.

### Investigations of ycf15 and ycf68

To investigate the distribution and nature of *ycf15*, we extracted the *ycf15 *sequence from the *Nicotiana tabaccum *genome (NC_001879) and conducted pairwise BLAST searches between this and each of the 63 then-available complete chloroplast genome sequences. If any portion of the *ycf15 *sequence was not detected in comparisons against whole genome sequences, the *ycf2:trnL *spacer region was extracted and just that portion of the genome was compared to the *Nicotiana ycf15 *sequence. ClustalW [75] alignments were also conducted to assess levels of conservation in a subset of genomes.

Pairwise BLAST (bl2seq [76]) was also used to assess *ycf68 *distributional patterns, here using the *ycf68 *sequence from *Zea *and *Nymphaea *against all 63 then-published plastid genomes. Also, since *ycf68 *is found in the *trnI*-GAU intron, it seemed possible that the conserved sequence could be related to folding during excision. The *trnI*-GAU intron was extracted using NCBI or DOGMA from all the species listed in Table 3 that contain an intron in *trnI*-GAU and folded using the web-based programs mfold [77] and DINAmelt [78] using default settings. We then examined the folding patterns to look for regions with conserved folding domains. We saw no obvious correlation between the position of the region of sequence similarity and the folding structure hypothesized by either program.

Another method for investigating the functionality of an ORF was designed by Echols *et al*. [31]. They looked at codon usage in pseudogenes, genes, and intergenic regions to determine how pseudogenes were evolving and if amino acid frequency could be an indicator of the functionality of a conserved region of DNA [31]. They separately calculated the frequency of amino acids in coding, noncoding, and known pseudogene sequences and then graphed the results to display trends of usage in the three sequence types. They found that the amino acid frequency in pseudogenes was an exact intermediate between amino acid frequency in known genes and intergenic DNA, and argued that this method is a valid way to determine whether or not a gene is functional [31]. We used this method to investigate whether or not *ycf15 *and *ycf68 *had amino acid frequencies similar to coding, noncoding, or an intermediate to differentiate among the hypotheses that the ycfs are genes, pseudogenes, or simply non-coding DNA. Algorithms from web-based suite of tools BABEL [79] were used to calculate the frequency of amino acids. For *Ranunculus *and *Nuphar*, we input all coding DNA sequences and recorded the amino acid frequency. We determined codon usage in the noncoding sequence by averaging the codon usage for each reading frame (the difference in frequency among the six reading frames is minimal) to get a frequency estimate. BABEL tools [79] were also used to calculate amino acid frequency in the intron sequence of *trnI*-GAU and the *ycf2:trnL*-CAA intergenic region. These results were then graphed. In the Echols *et al*. [31] analysis the amino acid frequency for each amino acid was graphed in order of decreasing levels of variation. We tried multiple methods for arranging the amino acids including that of Echols *et al*., alphabetical, high and low frequency, standard deviation of the frequency across all types of DNA, etc. We found that manipulation of the order of amino acids on the X-axis changed the results so much that we could display evidence for any of the hypotheses under consideration. Therefore, we do not believe that this type of analysis is valid, at least not with our data. Perhaps with the larger sample sizes, from nuclear genomes, available to Echols *et al*. the approach is more consistent.

We further investigated the characteristics of *ycf15 *and *ycf68 *by comparing sequence similarity of the *trnI*-GAU intron and the intergenic spacer between *ycf2 *and *trnL*-CAA to other introns and intergenic spacers throughout the chloroplast genome to see if the intergenic/intron regions containing *ycf15 *and *ycf68 *are more conserved than that of other noncoding DNA in the chloroplast genome. We used the web-based program Mulan [32] to compare noncoding DNA in our sequences to a reference sequence – *Nicotiana tabacum *for *ycf15 *and *Zea mays *for *ycf68*. Mulan performs pairwise sequence comparisons of the input sequence to the chosen reference sequence. We used the default setting of 100 for the ECR (evolutionary conserved region) length, the minimum amount of base pairs that have to align for similarity to register on the histogram, and 50 percent for the ECR similarity. The algorithm then returns graphical information about the likeness of the input sequences to the reference sequence; output is in the form of a histogram showing the similarity of the sequences from 50 to 100 percent. To see if introns were commonly conserved at a level equivalent to that represented by the *ycf68 *motif, we compared the intron in *trnI*-GAU with a sample of other introns within the inverted repeat (*trnA*-UGC and *ndhB*), and in the large single copy region (*trnG*-UCC, *trnL*-UAA, and *trnV-*UAC). To investigate conservation of the nucleotide sequence in the *ycf2*:*trnL *intergenic spacer, relative to the *ycf15 *question, we compared it to intergenic DNA that is outside of known operons in the inverted repeat (3'*rps12*:*trnV*-GAC and *trnN-*GUU:*ycf1*) and the large single copy region (*rpoB*:*trnC*-GCA, *trnT*-GGU:*psbD*, and *psaA*:*ycf3*).

### Codon Usage

Alternative start codon usage was determined based on published annotations with additional comparisons. All genes annotated with an alternative start were aligned to determine whether or not the start was homologous with non-alternative start codons in other taxa. If the annotated alternative start was the result of a longer or shorter annotation, the region homologous to the start codon of other taxa was examined to determine whether or not the normal start codon was present.

Codon usage patterns for each genome were determined using FREQSQ from BABEL [79] and CodonW [41]. The predicted relationship of ENc and GC3 was determined using Wright's equation as given by Novembre [43]. The equation as printed in the Wright 1990 paper [42] contains a typographical error.

### Nuphar-Nymphaea comparison

The complete chloroplast genome of *Nuphar *was sub-divided into individual coding and non-coding regions, and each region was compared against the entire *Nymphaea *genome using the BLAST2 algorithm [76] available on the National Center for Biotechnology Information website. The output files from BLAST2 were used to determine the percent similarity of each region. BLAST2 presents errors when one sequence under comparison contains mononucleotide repeats with runs greater than six nucleotides; n is indicated in place of each repeating nucleotide at those positions and considered "non-matching" in similarity calculations. These errors were checked against the genome sequences and corrected before calculations were made. Nucleotide sequence fragments that BLAST2 did not align (and did not show) were scored as zeros and negatively affected the % identity value. For example if only 425 of the known 450 nucleotides in a gene were aligned by BLAST2, and it was otherwise a perfect match, then the % identity would be 425/450 or 94.4%. BLAST2 does not display insertions or deletion events (indels) of greater than 10 nucleotides; such a disparity between the sequences would lead to the two adjoining regions of similarity being displayed as separate regions of identity.

We looked at indels as well as nucleotide similarity. We characterized indels as insertions or deletions relative to *Nuphar*. If *Nuphar *contained sequence for which there was no equivalent in *Nymphaea *is was considered an insertion, whereas if nucleotides in the *Nymphaea *sequence lacked a *Nuphar *equivalent it was a deletion. We used BLAST2 [76] to quantify indels in coding regions only. MULAN [32] was used to produce a total genome alignment for *Nuphar *and *Nymphaea*. Indels in the entire genome were quantified using this output. Simple subtraction of the coding region indels from the total number of indels provided the number found in intergenic regions. We quantified indels of 5–9 bp, 10–19 bp and 20 or greater bp.

### SSR Analysis

To locate and characterize SSRs in these genomes, we used the Simple Sequence Repeat (SSR) Extractor Utility [80]. We screened for all perfect mono-, di- and tri-nucleotide repeats of length at least 8,8, or 9 nucleotides, respectively. We also determined the number and nature of mono-, di- and tri- nucleotide repeats of length at least 10, 10 or 12 nucleotides. In addition to analyzing SSR content of *Nuphar *and *Ranunculus*, we examined the chloroplast genomes of the other completely sequenced land plant genomes available at the time of the analysis. These chloroplast genome sequences were obtained from GenBank (accession numbers given in Table 7). To assess possible relationships between the number of longer SSRs and the number of shorter SSRs as well as the number of SSRs and genome size and A+T-richness and frequencies of particular SSR motifs, we conducted Spearman rank correlation tests using an online calculator [55]. Predicted numbers of SSRs of particular composition were calculated based on the underlying compositional nucleotide frequencies. For example, if A+T frequency in the genome overall is 0.638 then the frequency of a mononucleotide repeat of length 8 nt would be (0.638)^8 ^and the number of length 8nt A+T SSRs expected would be the frequency* (genome length/SSR length). To determine whether the composition of SSRs deviated from expectation based on overall nucleotide, dinucleotide and trinucleotide frequencies of the genomes (determined using FREQSQ [79]), we used a Chi-square test. Predicted number of each class of repeat was calculated as the ((expected frequency of a particular type of repeat, calculated as just stated)/(total of expected frequencies of all categories)) * observed total number of repeats. For example, in *Huperzia*, which has an A+T frequency of 0.638 and a G+C frequency of 0.362, the expected frequency of A+T mononucleotide repeats of length 8 is (0.638)^8 ^and of G+C 8 nt mononucleotide SSRs is (0.362)^8^. The expected number of A+T mononucleotide runs, given that 147 mononucleotide repeats of 8nt and longer are observed in that genome, is (0.638)^8^/((0.638)^8^*(0.362)^8^)*147 = 145.44. We did simplify the calculations by basing our predictions on repeats of minimum length, which was also the mean and median length (i.e., we considered all the repeats that we observed to be of minimum length for these calculations).

### SDR analysis

Shared and unique direct and inverted repeats were identified for eight angiosperm chloroplast genomes using the Comparative Repeat Analysis program [81]. This program uses REPuter [82] but it has two additional features: it filters out repeats that are contained entirely within other repeats, and it identifies shared repeats among the input genomes by Blasting the repeats from each genome against all other genomes. For repeat identification, the following settings were used: (i) minimum repeat size of 30 bp; (ii) 90% or greater sequence identity, based on Hamming distance equal to 3; and (iii) an e-value of 2 for Blast comparisons against the other genomes.

## List of abbreviations

bp – base pair

COA – Correspondence Analysis of Codon Usage [41]

CpG (or GpC or ApT, etc) – to nucleotides adjacent on one DNA strand and thus linked by a phosphodiester bond (symbolized by the p)

DOGMA – Dual Organellar Genome Annotator [73]

ENc – Effective number of codons [42]

GC3 – percent G+C at the 3^rd ^position of codons

IGS – intergenic spacer

IR – inverted repeat

LSC – large single copy region

nt – nucleotide

RSCU – relative synonymous codon usage

S – "strong" nucleotide (G or C)

SDR – small dispersed repeat

SSC – small single copy region

SSM – slip strand mispairing

SSR – simple sequence repeat

W – "weak" nucleotide (A or T)

## Authors' contributions

LAR conceived of the project, drafted the manuscript, conducted the SSR, nucleotide composition and codon usage analyses and helped with and coordinated other aspects of the project; RP finished the *Ranunculus *sequence, completed the annotations of the two genomes, prepared the GenBank submissions, made the comparisons related to *ycf15 *and *ycf68*, compared SSRs of *Nuphar *and *Nymphaea*, compared *Nuphar *and *Ranunuclus *with other genomes for gene content, gene order and start codon usage, and drafted manuscript sections related to her work; TWC performed the finishing and initial annotation work on *Nuphar*, conducted experiments to confirm IR boundaries, and helped generate genome map figures; CD performed comparisons between *Nuphar *and *Nymphaea*, the initial COA analyses of codon usage in *Nuphar *and *Ranunculus*, and drafted the manuscript section related to the *Nuphar-Nymphaea *comparisons; HMF and JLB generated the draft sequence for the two genomes; RKJ assisted in the preparation of the sequencing templates, helped with the annotation of *Nuphar*, performed the SDR analyses, drafted the SDR manuscript section, and contributed to the design of the project. All authors assisted with manuscript preparation and read and approved the final draft.

## Supplementary Material

Additional file 1 Detailed comparison of SSRs. This table (Table S1) provides a more detailed comparison of SSRs among the plastid genomes of ten vascular plants (eight angiosperms, one gymnosperm and one pteridophyte). SSRs are enumerated by composition as well as length. Click here for file

Additional file 2 Comparison of SSRs between the plastomes of *Nuphar *and *Nymphaea*. This table (Table S2) shows the results of a comparison of each 10,10,12 SSR in the plastid genomes of *Nuphar *and *Nymphaea*. The nature of any difference in the SSR between the two genomes is given. Click here for file
