# Supplementary material for: Comparative chloroplast genomics: analyses including new sequences from the angiosperms Nuphar advena and Ranunculus macranthus
Source: BMC Genomics. 2007 Jun 15;8:174. doi: 10.1186/1471-2164-8-174 (PMC1925096; doi:10.1186/1471-2164-8-174)
Supplement: Additional file 1 — Detailed comparison of SSRs. This table (Table S1) provides a more detailed comparison of SSRs among the plastid genomes of ten vascular plants (eight angiosperms, one gymnosperm and one pteridophyte). SSRs are enumerated by composition as well as length. [file 1471-2164-8-174-S1.doc]

Table S1. More detailed comparison of SSRs among 10 land plant genomes.

| SSR abundance  by composition  and length (nt) | | *Huperzia* | *Pinus* | *Nuphar* | *Nymphaea* | *Amborella* | *Calycanthus* | *Ranunculus* | *Arabidopsis* | *Nicotiana* | *Triticum* |
| --- | --- | --- | --- | --- | --- | --- | --- | --- | --- | --- | --- |
| Mononucleotide Repeats | A 8-9 | 65 | 27 | 22 | 22 | 33 | 26 | 63 | 67 | 34 | 46 |
|  | A 10-11 | 11 | 5 | 8 | 3 | 9 | 13 | 9 | 20 | 6 | 4 |
|  | A 12+ | 1 | 3 | 5 | 4 | 9 | 1 | 4 | 9 | 5 | 6 |
|  | Total A | **77** | **35** | **35** | **29** | **51** | **40** | **76** | **96** | **45** | **56** |
|  | max A | 14 | 14 | 16 | 16 | 15 | 13 | 14 | 17 | 13 | 15 |
|  | C 8-9 | 3 | 4 | 3 | 2 | 2 | 2 | 3 | 2 | 1 | 3 |
|  | C 10-11 | 2 | 0 | 0 | 1 | 0 | 1 | 0 | 0 | 0 | 1 |
|  | C 12+ | 1 | 0 | 1 | 2 | 0 | 1 | 0 | 0 | 0 | 0 |
|  | Total C | **6** | **4** | **4** | **5** | **2** | **4** | **3** | **2** | **1** | **4** |
|  | max C | 12 | 9 | 15 | 13 | 8 | 12 | 9 | 8 | 8 | 10 |
|  | G 8-9 | 4 | 2 | 2 | 1 | 2 | 2 | 2 | 1 | 2 | 1 |
|  | G 10-11 | 1 | 0 | 1 | 0 | 0 | 0 | 0 | 0 | 0 | 0 |
|  | G 12+ | 1 | 0 | 1 | 0 | 0 | 0 | 0 | 1 | 0 | 0 |
|  | Total G | **6** | **2** | **4** | **1** | **2** | **2** | **2** | **2** | **2** | **1** |
|  | max G | 15 | 10 | 12 | 9 | 8 | 8 | 8 | 13 | 8 | 9 |
|  | T 8-9 | 50 | 21 | 22 | 23 | 29 | 40 | 48 | 95 | 41 | 42 |
|  | T 10-11 | 7 | 7 | 3 | 4 | 11 | 11 | 11 | 25 | 18 | 9 |
|  | T 12+ | 1 | 4 | 1 | 1 | 6 | 8 | 4 | 13 | 9 | 4 |
|  | Total T | **58** | **32** | **26** | **28** | **46** | **59** | **63** | **133** | **68** | **55** |
|  | max T | 12 | 17 | 14 | 13 | 14 | 15 | 16 | 17 | 17 | 15 |
|  | **Total, mono** | **147** | **73** | **69** | **63** | **101** | **105** | **144** | **233** | **116** | **119** |
| Dinucleotide Repeats | WW 8 or 10 | 16 | 12 | 37 | 38 | 16 | 11 | 31 | 11 | 25 | 14 |
|  | WW 12 or 14 | 4 | 2 | 4 | 0 | 1 | 3 | 2 | 3 | 0 | 1 |
|  | WW 16+ | 2 | 0 | 3 | 0 | 3 | 1 | 1 | 1 | 0 | 0 |
|  | Total WW | **22** | **14** | **44** | **38** | **20** | **15** | **34** | **15** | **25** | **15** |
|  | max WW | 18 | 14 | 22 | 10 | 18 | 16 | 16 | 16 | 10 | 12 |
|  | WS/SW 8 or 10 | 12 | 18 | 19 | 22 | 27 | 20 | 24 | 20 | 16 | 18 |
|  | WS/SW 12 + | 0 | 0 | 0 | 0 | 0 | 0 | 0 | 0 | 0 | 0 |
|  | Total WS/SW | **12** | **18** | **19** | **22** | **27** | **20** | **24** | **20** | **16** | **18** |
|  | SS | **0** | **0** | **0** | **0** | **0** | **0** | **0** | **0** | **0** | **0** |
|  | **Total, di** | **34** | **32** | **63** | **60** | **27** | **35** | **58** | **35** | **41** | **33** |

| Trinucleotide Repeats | SSS 9 | 1 | 1 | 0 | 0 | 0 | 0 | 0 | 0 | 0 | 0 |
| --- | --- | --- | --- | --- | --- | --- | --- | --- | --- | --- | --- |
|  | SSS 12+ | 0 | 0 | 0 | 0 | 0 | 0 | 0 | 0 | 0 | 0 |
|  | Total SSS | 1 | 1 | 0 | 0 | 0 | 0 | 0 | 0 | 0 | 0 |
|  | 2S,1W 9 | 8 | 8 | 11 | 10 | 8 | 11 | 10 | 4 | 13 | 3 |
|  | 2S,1W 12 | 0 | 0 | 1 | 0 | 0 | 0 | 0 | 0 | 0 | 0 |
|  | 2S,1W 15 | 0 | 0 | 0 | 0 | 0 | 0 | 0 | 0 | 0 | 0 |
|  | Total 2S,1W | **8** | **8** | **12** | **10** | **8** | **11** | **10** | **4** | **13** | **3** |
|  | 2W,1S 9 | 35 | 23 | 39 | 34 | 33 | 26 | 24 | 38 | 38 | 31 |
|  | 2W,1S 12 | 1 | 0 | 3 | 1 | 0 | 1 | 1 | 3 | 2 | 1 |
|  | 2W,1S 15 | 2 | 0 | 0 | 0 | 0 | 0 | 0 | 2 | 0 | 0 |
|  | Total 2W, 1S | **38** | **23** | **42** | **35** | **33** | **27** | **25** | **43** | **40** | **32** |
|  | Max 2W,1S | 15 | 9 | 12 | 12 | 9 | 12 | 12 | 15 | 12 | 12 |
|  | WWW 9 | 30 | 10 | 22 | 24 | 10 | 13 | 17 | 14 | 17 | 6 |
|  | WWW 12 | 2 | 0 | 6 | 3 | 5 | 3 | 1 | 3 | 3 | 1 |
|  | WWW 15+ | 1 | 0 | 0 | 0 | 0 | 0 | 1 | 2 | 0 | 1 |
|  | Total WWW | **33** | **10** | **28** | **27** | **15** | **16** | **19** | **19** | **20** | **8** |
|  | Max WWW | 30 | 9 | 12 | 12 | 12 | 12 | 15 | 15 | 12 | 15 |
|  | **Total-tri** | **80** | **41** | **82** | **72** | **56** | **54** | **54** | **66** | **73** | **43** |
|  | mono | 147 | 73 | 69 | 63 | 101 | 105 | 144 | 233 | 116 | 119 |
|  | di | 34 | 32 | 63 | 60 | 27 | 35 | 58 | 35 | 41 | 33 |
|  | tri | 111 | 41 | 82 | 72 | 56 | 54 | 54 | 66 | 73 | 43 |
|  | **TOTAL** | **292** | **146** | **214** | **195** | **184** | **194** | **256** | **334** | **230** | **195** |

“Max” gives the maximum repeat length (in nucleotides) of that motif and is included where the maximum length cannot be inferred from the other data reported. The IUPAC ambiguity codes, “W” and “S”, are used to indicate “A and/or T” and “G and/or C”, respectively.
