# Supplementary material for: Comparative chloroplast genomics: analyses including new sequences from the angiosperms Nuphar advena and Ranunculus macranthus
Source: BMC Genomics. 2007 Jun 15;8:174. doi: 10.1186/1471-2164-8-174 (PMC1925096; doi:10.1186/1471-2164-8-174)
Supplement: Additional file 2 — Comparison of SSRs between the plastomes of Nuphar and Nymphaea. This table (Table S2) shows the results of a comparison of each 10,10,12 SSR in the plastid genomes of Nuphar and Nymphaea. The nature of any difference in the SSR between the two genomes is given. [file 1471-2164-8-174-S2.doc]

Table S2. Comparison of each 10,10,12 SSRs between the plastomes of *Nuphar* and *Nymphaea*.

| Repeat  Motif | Type of difference(s)^a^ | *Nuphar* (number repeats) | *Nymphaea* (number of repeats) | Repeat Position^b^ | |
| --- | --- | --- | --- | --- | --- |
|  |  |  |  | IGS | intron |
| A | none | 16 | 16 | S:G |  |
| A | point | 15 | 7 |  | rps16 |
| A | SSM | 14 | 10 | T:psbD |  |
| A | point | 13 | 7 | rps16:Q |  |
| A | SSM,point | 12 | 6 | ndhF:rpl23 |  |
| A | point | 11 | 5 | trnK:rps16 |  |
| A | SSM | 11 | 5 | psbM:D |  |
| A | point | 10 | 9 |  | clpP,intron 1 |
| A | SSM | 10 | 9 | C:petN |  |
| A | point | 10 | 7 | rps16:Q |  |
| A | SSM | 10 | 5 | trnK:rps16 |  |
| A | point | 9 | 14 | P:psaJ |  |
| A | point | 6 | 14 |  | clpP,intron 2 |
| A | SSM,indel | 6 | 12 | atpH:atpI |  |
| A | SSM | 6 | 10 | L:ccsA |  |
| AAC | point | 3 | 3 | ndhE:ndhG |  |
| AAG | none | 4 | 4 | rps7:V |  |
| AAG | none | 3 | 3 | K:rps16 |  |
| AAG | none | 3 | 3 |  | ycf3,intron2 |
| AAG | point | 3 | 3 | T:L |  |
| AT | SSM | 9 | 4 | S:G |  |
| AT | point,indel | 6 | 3 | accD:psaI |  |
| AT | none | 5 | 5 | rpoB:C |  |
| AT | point | 5 | 4 | psaI:ycf4 |  |
| AT | SSM | 5 | 4 | atpB:rbcL |  |
| AT | SSM | 5 | 3 | rbcL:accD |  |
| AT | SSM | 4 | 5 | psaI:ycf4 |  |
| AT,TA | SSM | 4 | 5 | atpB:rbcL |  |
| C | SSM,indel | 6 | 13 | psbZ:G |  |
| CAT | none | 3 | 3 |  | rps12 |
| CTC | SSM | 3 | 1 | T:psbD |  |
| CTT | none | 3 | 3 | F:ndhJ |  |
| G | SSM,point | 12 | 6 | psaI:ycf4 |  |
| G | SSM | 11 | 4 | petA:psbJ |  |
| G | none | 9 | 9 |  | trnI |
| GAA | point | 3 | 3 | psaJ:rpl33 |  |
| GAT | none | 3 | 3 |  | rpl2 |
| GGA | none | 3 | 3 | infA:rps8 |  |
| GT | none | 4 | 4 | psbZ:G |  |
| GTT | none | 3 | 3 | ycf3:S |  |
| GTT | SSM | 3 | 2 | rpoB:C |  |
| T | SSM | 8 | 10 | ndhF:rpl23 |  |
| T | SSM,indel | 7 | 10 | psaC:ndhE |  |
| T | SSM | 4 | 10 | rps19:rpl2 |  |
| TA | point | 6 | 4 | S:psbZ |  |
| TA | SSM | 6 | 4 | P:psaJ |  |
| TA | point,indel | 5 | 5 | ndhC:V |  |
| TA | point | 5 | 4 | ndhG:ndhI |  |
| TAT | none | 4 | 4 | N:ndhF |  |
| TAT | SSM | 4 | 1 |  | trnL |
| TAT | point,indel | 3 | 4 | accD:psaI |  |
| TCC | SSM,point | 1 | 3 | T:psbD |  |
| TCT | point | 4 | 3 | V:rps12_3' |  |
| TGT | point | 3 | 2 | rps16:Q |  |
| TTC | none | 4 | 4 | psaJ:rpl33 |  |

^a^SSM stands for slip-strand mispairing and indicates a difference in repeat unit number. In contrast, a difference termed an “indel” if there was a difference in length that did not correspond to a difference in repeat unit number.

^b^All SSRs that exceeded 10 nt in length (for mono: and di: nucleotide repeats) or 12 nt (for trinucleotide repeats) in either genome existed in non-coding regions, either intergenic spaces (IGS) or introns. Single letter gene designations indicate tRNA genes.
